# Systems Approach Reveals Nuclear Factor Erythroid 2-Related Factor 2/Protein Kinase R Crosstalk in Human Cutaneous Leishmaniasis

**DOI:** 10.3389/fimmu.2017.01127

**Published:** 2017-09-15

**Authors:** Áislan de Carvalho Vivarini, Teresa Cristina Calegari-Silva, Alessandra Mattos Saliba, Viviane Sampaio Boaventura, Jaqueline França-Costa, Ricardo Khouri, Tim Dierckx, Karina Luiza Dias-Teixeira, Nicolas Fasel, Aldina Maria Prado Barral, Valéria Matos Borges, Johan Van Weyenbergh, Ulisses Gazos Lopes

**Affiliations:** ^1^Laboratory of Molecular Parasitology, Carlos Chagas Filho Biophysics Institute, Center of Health Science, Federal University of Rio de Janeiro, Rio de Janeiro, Rio de Janeiro, Brazil; ^2^Department of Microbiology, Immunology and Parasitology – FCM/UERJ, State University of Rio de Janeiro, Rio de Janeiro, Rio de Janeiro, Brazil; ^3^Integrated Laboratory of Microbiology and Immunoregulation, Gonçalo Moniz Research Center, Oswaldo Cruz Foundation, Salvador, Bahia, Brazil; ^4^Department of Microbiology and Immunology, Rega Institute for Medical Research, KU Leuven, Leuven, Belgium; ^5^Faculty of Biology and Medicine, Department of Biochemistry, University of Lausanne, Lausanne, Switzerland

**Keywords:** *Leishmania*, macrophage, nuclear factor erythroid 2-related factor 2, PKR, Sod1

## Abstract

*Leishmania* parasites infect macrophages, causing a wide spectrum of human diseases, from cutaneous to visceral forms. In search of novel therapeutic targets, we performed comprehensive *in vitro* and *ex vivo* mapping of the signaling pathways upstream and downstream of antioxidant transcription factor [nuclear factor erythroid 2-related factor 2 (Nrf2)] in cutaneous leishmaniasis (CL), by combining functional assays in human and murine macrophages with a systems biology analysis of *in situ* (skin biopsies) CL patient samples. First, we show the PKR pathway controls the expression and activation of Nrf2 in *Leishmania amazonensis* infection *in vitro*. Nrf2 activation also required PI3K/Akt signaling and autophagy mechanisms. Nrf2- or PKR/Akt-deficient macrophages exhibited increased levels of ROS/RNS and reduced expression of *Sod1* Nrf2-dependent gene and reduced parasite load. *L. amazonensis* counteracted the Nrf2 inhibitor Keap1 through the upregulation of p62 *via* PKR. This Nrf2/Keap1 observation was confirmed *in situ* in skin biopsies from *Leishmania*-infected patients. Next, we explored the *ex vivo* transcriptome in CL patients, as compared to healthy controls. We found the antioxidant response element/Nrf2 signaling pathway was significantly upregulated in CL, including downstream target p62. *In silico* enrichment analysis confirmed upstream signaling by interferon and PI3K/Akt, and validated our *in vitro* findings. Our integrated *in vitro, ex vivo*, and *in silico* approach establish Nrf2 as a central player in human cutaneous leishmaniasis and reveal Nrf2/PKR crosstalk and PI3K/Akt pathways as potential therapeutic targets.

## Introduction

Human cutaneous leishmaniasis (CL) is spread worldwide, and the incidence is estimated to be from 0.7 to 1.2 million cases each year (1). Different clinical manifestations occur in humans due to the immune response and the infection by distinct *Leishmania* species (2). *Leishmania* parasites exhibit a plethora of adaptive mechanisms that interfere with several macrophage functions through the manipulation of host signaling pathways (3).

The imbalance between oxidative stress and cytoprotective systems of detoxification dictates the outcome of intracellular parasitic infections (4). The transcription factor [nuclear factor erythroid 2-related factor 2 (Nrf2)] is a master regulator of phase II defense gene expression that may protect cells from oxidative stress. The DNA promoter sequences of phase II defense genes share the canonical antioxidant response element (ARE), which is recognized by Nrf2 (5). Nrf2-dependent expression leads to profound effects on the suppression of the inflammatory response and immune activation through *Toll*-like receptors (6, 7).

The control of Nrf2 activation is dictated by different post-translational modifications. Multiple sites on the Nrf2 protein are phosphorylated by kinases, such as PERK, members of the MAPK family, PKC_ζ_, and GSK3β, increasing the nuclear translocation and binding of the protein to ARE elements on the promoters of target genes (8–10).

The PI3K/Akt pathway modulates Nrf2 signaling (11) and, importantly, recent reports have demonstrated the modulation of the PI3K/Akt pathway upon *Leishmania amazonensis* infection (12). The enzyme GSK3, a target of Akt1, phosphorylates the Nh6 domain of Nrf2 and facilitates the action of ubiquitin ligase, leading to proteasomal degradation. The inhibition of GSK3 by phosphorylation allows the nuclear translocation of Nrf2 (13).

Kelch-like ECH-associated protein 1 (Keap1) is a major inhibitor of Nrf2 that constitutively induces the ubiquitination of the Nh2 domain, directing Nrf2 to proteasomal degradation. Keap1 is uncoupled from Nrf2 because of post-translational modifications due to oxidative stress, releasing Nrf2 for nuclear translocation (14).

Autophagy may modulate Nrf2 activation *via* Keap1 degradation (15). The components of the autophagy pathway are sensors of oxidative stress (16), and the increase in the expression of *p62* (*Sqstm1*), an Nrf2 target autophagy gene (17), favors the cellular capacity to process proteins destined for the autophagosome, decreasing oxidative stress (18).

The phosphorylation of p62 allows its binding to several cargo proteins, including Keap1, leading to autophagy as well as the Nrf2 stability and activation (19). The activation of Nrf2 through the p62/autophagy non-canonical pathway has also been demonstrated in macrophages treated with LPS, PolyI:C and peptidoglycan (PGN) upon the engagement of TLR4, TLR3, and TLR2, respectively (20).

Double-stranded RNA-dependent protein kinase [protein kinase R (PKR)] has in the Nh2-terminal domain two double-stranded RNA-binding motifs, and its kinase catalytic domain is located in the carboxyl-terminal (21, 22). On binding dsRNA, PKR dimerizes and undergoes autophosphorylation at multiple sites (23). Expression of catalytically defective mutant PKR (K296R) in cells inhibited the autophosphorylation and subsequent the activation of its major substrate eIF2-α (24, 25). PKR-mediated signaling may promote autophagy through eIF2-α phosphorylation (26). In several viral infections, PKR plays an essential role in the autophagy trigger (27). In STAT3^−/−^ cells, PKR is able to induce autophagy through LC3-I to LC3-II conversion and the formation of vacuole compartments (28). In cells treated with type I interferon (IFN-I), both LC3 activation and p62 expression are increased (29).

In this work, we unveil the mechanisms that regulate Nrf2 gene expression in a PKR-dependent fashion. We describe for the first time the signaling pathway that coordinates Nrf2 activation during *Leishmania* infection. Finally, the induction of cytoprotective genes through the novel PKR/Nrf2 pathway may represent a prominent therapeutic mechanism for treatment and guide the development of novel targets in both infectious and inflammatory diseases.

## Materials and Methods

### Reagents

Chloroquine diphosphate salt, dl-sulforaphane (SFN), phorbol-12 myristate-13 acetate (PMA), *N*-acetyl-l-cysteine (NAC), Wortmannin, and LY294002 hydrochloride were purchased from Sigma-Aldrich (St. Louis, MO, USA). AKTi (AKT inhibitor VIII, Akt1/2) was purchased from Santa Cruz Biotechnology (Dallas, TX, USA). Poly (cytidylic-inosic) acid potassium salt (PolyI:C) and the PKR inhibitor CAS 608512-97-6 were purchase from Calbiochem-Millipore (Darmstadt, Germany). Human recombinant interferon-alpha 2b was obtained from Blausiegel (Cotia, SP, Brazil).

### Cell Lines and Culture

The mouse macrophage leukemia cell line RAW 264.7 (TIB-71; American Type Culture Collection (ATCC), Manassas, VA, USA), the human monocytic leukemia cell line THP-1 (ATCC:TIB202TM) and the human embryonic kidney cell line HEK-293T (ATCC:CRL-11268) were maintained in DMEM medium with high glucose (Vitrocell Embriolife, Campinas, SP, Brazil) supplemented with 10% heat-inactivated fetal bovine serum (Sigma-Aldrich, St. Louis, MO, USA). THP-1 cells were differentiated to macrophages with 40 ng/mL of PMA for 3 days. Afterward, the cells were washed three times with PBS and incubated with fresh medium for an additional 3 days. RAW 264.7 cells expressing either empty vector (RAW-Bla cells) or a dominant-negative PKR K296R (RAW-DN-PKR cells) were donated by Dr. Aristóbolo Silva, Federal University of Minas Gerais, Brazil.

### Peritoneal Macrophages

Ten-week-old male 129/SvEv PKR^−/−^ (PKR-ko) and their respective wild-type littermates (WT) were used for experiments. Briefly, 4 days before peritoneal lavage, 2 mL of 3% thioglycolate were intra-peritoneally injected in each mouse. Thioglycolate-elicited peritoneal macrophages from wild-type or PKR-knockout 129Sv/Ev were obtained by injecting 8 mL of serum-free DMEM into the peritoneal cavity. After 1 h, the cells were washed once in PBS and then plated in in DMEM medium supplemented with 10% FBS on glass coverslips at 2 × 10^5^/well in 6-well or 24-well polystyrene plates for subsequent *Leishmania* infection assays.

### Cell Treatment

To induce the activation of Nrf2, 10 mM SFN were used as positive controls. For the inhibition of PKR activity, we pretreated the cells for 1 h with 300 nM of the PKR inhibitor (PKRi). To induce PKR activation, poly(inosinic-cytidylic-) acid potassium salt (PolyI:C) at a final concentration of 25 µg/mL or recombinant IFNα-2b at 1,000 U/mL were used. PI3K/Akt inhibition was accomplished by cell treatment with 10 µM LY294002, 10 mM Wortmannin or 5 mM AKTI (AKT inhibitor VIII Akt1/2). To inhibit autophagy, we used 40 µM chloroquine. *N*-acetylcysteine (NAC) was used at a concentration of 10 mM.

### Parasites, Culture Conditions, and Infection

*Leishmania (Leishmania) amazonensis* (WHOM/BR/75/Josefa) and *Leishmania (Viannia) braziliensis* (BA788) were used in this study. The *L. (L.) amazonensis* strains obtained from biopsies of patients with diffuse cutaneous leishmaniasis (DCL) (Ba276, Ba336, and Ba760) or localized cutaneous leishmaniasis (Ba69, Ba73, and Ba125) were also used *in vitro* assays. The promastigote forms were grown at 26°C in Schneider’s Insect Medium (Sigma-Aldrich) with 10% fetal bovine serum, and metacyclic promastigotes were collected from stationary cultures and used for cell infections. Macrophages were infected with *Leishmania* promastigotes at a parasite:cell ratio of 10:1 at 37°C. Infected macrophages were counted in a Neubauer Chamber by light microscopy to assess the infection index, which was calculated by multiplying the percentage of infected macrophages by the average number of parasites per macrophage in Giemsa-stained slides.

### Immunoblotting

THP-1 cells (1 × 10^6^ cells) were washed twice with ice-cold PBS and then lysed in 100 µL of lysis buffer (50 mM Tris-HCl, pH 7.5, 5 mM EDTA, 10 mM EGTA, 50 mM NaF, 20 mM β-glycerophosphate, 250 mM NaCl, 0.1% Triton X-100, 1 µg/mL BSA, and a 1:100 dilution of protease inhibitor cocktail, Sigma-Aldrich, St. Louis, MO, USA) for total protein extraction. For nuclear protein extraction, after infection and/or treatment, the cells were washed twice with 1x PBS and then lysed with 100 µL of buffer A (HEPES 10 mM pH 7.9. 10 mM KCl, 0.1 mM EDTA, 0.1 mM EGTA, NP-40 0,25% (v/v); cocktail of protease inhibitors) for 10 min on ice. The lysed cells were centrifuged at 14,000 *g* for 1 min at 4°C, and the pellet was resuspended in 60 µL of buffer C (20 mM HEPES pH 7.9, 0.4 M NaCl, 1 mM EDTA, 1 mM EGTA, 20% glycerol, protease inhibitor cocktail) and incubated on ice for 20 min. The lysate was centrifuged at 14,000 *g* for 5 min, and the supernatant containing nuclear proteins was collected in a new tube. The protein extracts were subjected to electrophoresis on 10% SDS-polyacrylamide gels and transferred to nitrocellulose membranes (Amersham Biosciences, Piscataway, NJ, USA). After blocking with 5% non-fat dry milk in TBS with 0.1% Tween-20 (TBS-T), the blots were incubated over-night with antibodies against PKR (12297), Nrf2 (12721), GSK3 (9369), Sqstm1/p62 (5114), LC3B (2775), phospho-GSK3β-Ser9 (9336), phospho-Akt-Ser473 (9271), phospho-eIF2α-Ser51 (9721), α-Tubulin (2144), β-Tubulin (2146), and Lamin A/C (2032) from Cell Signaling Technology; phospho-PKR Th451 (07-886) from Millipore; keap1 (150654) from Abcam; and β-actin (47778), Sod1 (8637), followed by anti-rabbit (2004) or anti-mouse (2005) horseradish peroxidase-conjugated IgG (1:4,000) from Santa Cruz Biotechnology. The membranes were then submitted to three washes with 0.1% TBS-T after each incubation, and the proteins were detected using the ECL chemiluminescent detection system (Amersham Biosciences).

### Immunohistochemistry

To validate the differential expression of Nrf2 (C20—Santa Cruz Biotechnology) and keap1 (150654—Abcam) in DCL and LCL samples, immunohistochemistry was performed on formalin-fixed, paraffin-embedded (FFPE) sections. Briefly, after deparaffinization, rehydration and target retrieval (DAKO Corporation, Hamburg, Germany), slides from five DCL and five LCL cases were incubated with serum-free protein block reagent and then incubated overnight with Nrf2 or Keap1 (4and 10 mg/mL, respectively, both from Abcam, Cambridge, United Kingdom) or anti-rabbit isotype control antibodies. After the sequential application of a peroxidase-blocking reagent, DAKO EnVision + System-HRP (DAKO Corporation, Hamburg, Germany), digital images of the tissue sections were captured using a Nikon E600 light microscope and a Q-color 1 Olympus digital camera. Sections of prostate and lung adenocarcinoma were used as positive controls. Quantification of the stained areas was performed using Image Pro Plus software (Media Cybernetics).

### Luciferase Assays

To investigate the promoter activity, RAW-264.7 cells (1 × 10^5^ cells per well) was plated in 48-well polystyrene plates and transfected with 1 µg of reporter plasmids using LIPOFECTAMINE 2000 reagent (Invitrogen, Carlsbad, CA, USA). THP-1 cells (2 × 10^6^) were transfected with 0.5 µg of luciferase reporter plasmids using Nucleofector™ Technology (Lonza, Basel, Switzerland) according to the manufacturer’s instructions. The following plasmids were employed in the assays: Sod1-basal, Sod1-ΔARE, Sod1-WT, 3xARE, and Nrf2-WT. For normalization of the luciferase readout, the plasmid pRL-CMV (Promega) was used. After infection and treatment, the cells were washed with PBS, lysed according to the Dual Luciferase System protocol (Promega), and analyzed using the GloMax^®^-Multi detection system (Promega Corp., Madison, WI, USA).

### Chromatin Immunoprecipitation Assay (ChIP)

Chromatin immunoprecipitation assay analysis was carried out according to the Simple ChIP Enzymatic Chromatin IP kit protocol (Cell Signaling). RAW 264.7 (WT-PKR and DN-PKR) cells or the human monocytic leukemia cell line THP-1 (ATCC:TIB202TM) were plated to confluence in 15 cm dishes. After infection, the cells were fixed with 1% formaldehyde for 10 min at room temperature, followed by the addition of glycine to a final concentration of 125 mM for 5 min at room temperature prior to cell lysis. One unit of micrococcal nuclease was added to the sample and incubated for 20 min at 37°C to digest DNA to the length of approximately 150–800 base-pairs. The chromatin was immunoprecipitated with 5 µg/mL anti-Nrf2 antibody (D1Z9C-XP—Cell Signaling Technology, Danvers, MA, USA) at 4°C under rotation for 16 h. The DNA isolated from the immunoprecipitated material was amplified by real-time PCR using SybrGreen, and the DNA sequences of the primers used were Sod1-ARE.chip-F: 5′-AAGTCCGGGTCCCAGCTCAGAG-3′ and Sod1-ARE.chip-R: 5′-TTGGTGCAAGCACACCGGGAG-3′; p62-ARE.chip-F: 5′-CCCCACAGTTCCCCATTGGC-3′ and p62-ARE.chip-R: 5′-GACAGTGGGGACGCAAAGGC-3′; and Nrf2-AREL2chip-F: 5′-AAGTCCGGGTCCCAGCTCAGAG-3′ and Nrf2-AREL2chip-R: 5′-TTGGTGCAAGCACACCGGGAG-3′. As a control, 1/50 of the digested input chromatin was similarly processed and analyzed in the absence of immunoprecipitation. To calculate the input percentage of the samples, the input was adjusted to 100% (average Ct of input − Log_2_ of 50), followed by the application of the 100 × 2^(adjusted input − average Ct(IP))^ formula.

### Cloning and Generation of Luciferase Reporter Plasmids

Total DNA was extracted from THP-1 cells using a Wizard^®^ Genomic DNA Purification kit (Promega) and measured using a BioPhotometer (Eppendorf). One PCR was carried out with primers spanning different regions of the *Sod1* and *Nrf2* promoters, yielding different fragment sizes, in the following conditions: 20 ng of genomic DNA and 35 cycles of 95°C for 1 min, 58°C for 1 min, and 72°C for 1 min. The DNA sequences of the primers used were Sod1.wt-F: 5′- GTCTCGAGCTGTAGGGTTGTGGCCTTGCCAAA-3′, Sod1.ΔARE-F: 5′-GTCTCGAGGCCAATTTCGCGTACTGCAACCG-3′, Sod1.basal-F: 5′-GTCTCGAGCTCGCGACCCGAGGCTG-3′ and Sod1-R: 5′-GTAGATCTCAGGAGACTACGACGCAAACCAGC-3′; and Nrf2-F: 5’ AAGTCCGGGTCCCAGCTCAGAG 3′ and Nrf2-R: 5′-TGGGGGCGGAACAAGGACCTAG-3′. A 1.8% agarose gel was run for 50 min at 100 V, and the amplicons were extracted from the gel and purified with the Zymoclean Gel DNA Recovery kit ™ (Zymo Research). The amplicons were ligated into a pJet-Blunt plasmid (Fermentas) with T4 ligase (Promega) for the first selection of positive colonies. After confirming positivity through PCR and a digestion assay, a colony was selected and grown, and a new plasmid extraction was performed. Digestion of the pJet-Blunt vector containing subcloned amplicons was performed with the Bgl-II enzyme (Promega), and the products were subjected to electrophoresis on a 2% agarose gel to extract the gel fragments. The pGL2-basic plasmid was also digested with the Bgl-II enzyme for the subsequent binding of the amplicons with T4 ligase enzyme (Promega). The cloned fragments and final vectors were then transformed into DH5α bacteria, and colonies were selected for further confirmation by sequencing. To obtain a luciferase-expressing pGL2-basic plasmid containing three copies of the sequence regulatory region ARE (3xARE), two oligos (5′-ATGCCGCTCGAGAATGACATTTCTAGAATGACATTTCTAGAATGACATTTCTAGAGATCTCGGCCG-3′ and 3′-TACGGCGAGCTCTTACTGTAAAGATCTTACTGTAAAGATCTTACTGTAAAGATCTCTAGAGCCGGC-5′) were designed and annealed to serve as templates for a PCR under the following conditions: 20 ng of DNA oligo and 35 cycles of 95°C for 1 min, 52°C for 1 min, and 72°C for 1 min with the primers 3xARE-F: 5′-ATGCCGCTCGAGAATG 3′, and 3xARE-R: 5′-CGGCCGAGATCTCTAGA 3′. The binding reactions and digestion with the Bgl-II enzyme followed the same protocol as described above.

### Lentiviral Production and THP-1 Transduction

HEK-293T cells were used for shNrf2 lentiviral production. Initially, we co-transfected the cells with two packaging plasmids (pΔ8.9 and pVSVG) containing accessory proteins for the generation of the virus and capsid, respectively, along with the plasmid pLKO.1-shMission-Nrf2 (Sigma-Aldrich). For HEK-293T transfection, 60 µL of FuGENE HD reagent (Promega) was used in a 100 mm dish containing approximately 4 × 10^6^ cells, along with 10 µg of target plasmid, 6 µg of pVSVG and 4 µg of pΔ8.9. After 24 h of transfection, the culture medium was changed and, over the next 2 days, the supernatants were collected at 10 mL/day. The 20 mL of supernatant was ultracentrifuged at 16,000 rpm for 90 min at 4°C, and the pellet was resuspended in 1 mL of DMEM without serum. Viral transduction in THP-1 cells was accomplished in 2 × 10^6^ cells incubated with 1 mL of virus preparation for 48 h.

### Fluorimetric Assays

The production of reactive oxygen species (ROS), nitric oxide (NO), and peroxynitrite (OONO) was performed by fluorimetry. For this, 10^5^ cells were seeded in black 96-well plates and maintained for 24 h in DMEM containing 10% fetal bovine serum. The day after, the cells were washed three times with PBS, and HBSS medium without serum was added and incubated for 1 h at 37°C and 5% CO_2_. The cells were incubated with different fluorescent probes for 1 h. Then, the cells were washed with PBS and treated with medium or infected with *L. amazonensis*. Fluorescence counting was monitored after incubation at 1-h intervals for up to 6 h (GloMax™). The production of ROS was detected using the probe CM-H2DCFDA (5 mM, Molecular Probes), with excitation at 495 nm and emission at 525 nm. For NO production, the DAF-FM probe (5 mM, Molecular Probes) was used, with excitation at 495 nm and emission at 515 nm. For the production of OONO^-^, the probe HPF (5 mM, molecular probes) was used, with excitation at 490 nm and emission at 515 nm.

### Patient Characteristics

Diffuse cutaneous leishmaniasis patients (*n* = 4) were recruited at our reference clinic in São Luiz, Maranhão, Brazil. DCL patients exhibited chronic progression of the disease with several remissions, multiple nodular and highly parasitized lesions throughout the skin, and a negative DTH response. LCL patients (*n* = 5), recruited at our reference clinic in Jiquiriçá, Bahia, Brazil, had a single or a few ulcerated lesions present for up to 2 months and a positive DTH response (30). The clinical and epidemiological data from patients with DCL and those with LCL are summarized in Table S3 in Supplementary Material. Skin biopsies were preserved as paraffin-embedded specimens.

### Patient Recruitment and Diagnosis for Transcriptomic Analysis

This study was approved by the Ethics Committee of the Gonçalo Moniz Research Center (FioCruz-Bahia). Informed consent was obtained from all patients and healthy controls. CL patients were diagnosed according to characteristic lesion morphology, positive skin test, seropositivity toward *Leishmania* antigen and/or the presence of parasites in the lesion. LCL patients infected with *Leishmania braziliensis* (*n* = 18, 10 male, 29.6 ± 2.3 years) were recruited at diagnosis (before treatment) in two outpatient clinics (Jequié and Jiquiriçá-BA, NE Brazil) covering the same rural area.

### Ethics Statement

Written informed consent was obtained from all participants or legal guardians, and all of the data analyzed were anonymized. The project was approved by the Institutional Review Board of Centro de Pesquisas Gonçalo Moniz, FIOCRUZ–BA (license number 136/2007) and complies with the guidelines of the Declaration of Helsinki.

### Microarray Analysis

PBMCs from LCL patients and healthy controls were processed in parallel and immediately frozen in Trizol to preserve RNA integrity. Following Trizol extraction, total RNA was further purified using an RNeasy kit according to the manufacturer’s protocol (QIAGEN, Venlo, Netherlands). Affymetrix Whole Genome microarray analysis was performed by the VIB MicroArray Facility (Leuven, Belgium) using a GeneChip^®^ Human Gene 1.0 ST Array with the WT PLUS reagent kit (Affymetrix, Santa Clara, CA, USA) according to the manufacturer’s specifications. Data preprocessing (RMA) was performed using the Bioconductor xps package. Microarray data were deposited in GEO (accession number: GEO Submission (GSE80008) (NCBI tracking system #17832057)).

### nCounter Digital Transcriptomics

RNA extraction from skin biopsies was performed as above. Digital quantification of selected genes (NRF2, PKR, SOD1, SOD2, KEAP1, HMOX1) was performed by nCounter (Nanostring). Two-step normalization using internal positive and negative control RNAs, as well as PTPRC (CD45) normalization to correct for differences in tissue leukocyte infiltration, was performed as previously described (31).

### Enrichment Analysis

The ingenuity pathway analysis (IPA) program was used to perform the initial pathway/function level analysis on genes determined to be differentially expressed in the microarray analysis (IPA version 9.0, Build 116623, Content version 3211, Ingenuity Systems, Red Wood City, CA, USA). Uncorrected *p*-values and absolute fold-changes were used with cut-offs of *p* < 0.05. Based on a scientific literature database, the genes were sorted into gene networks and canonical pathways, and significantly overrepresented pathways were identified. Further enrichment analysis was performed, including Gene Ontology (GO) term enrichment using the WEB-based GEne SeT AnaLysis Toolkit (WebGestalt), KEGG pathway enrichment using the pathway database from the Kyoto Encyclopedia of Genes, and Genomes and transcription factor target enrichment using data from the Broad Institute Molecular Signatures Database (MSigDB). Genesets from the GO, KEGG pathways, WikiPathways, and Pathway Commons databases, as well as transcription factors, were considered overrepresented if their corrected *p*-value was smaller than 0.05.

### Statistical Analysis

The data were analyzed by one-way ANOVA for independent samples or Mann–Whitney (two-sided *t*-test) using Prism 5 software. The data represent the mean ± SD of the mean. The data are expressed as the average of three independent determinations, and significant differences were indicated by *p < 0.05.

## Results

### *Leishmania* Induces Nrf2 *via* PKR

**Figure 1:**
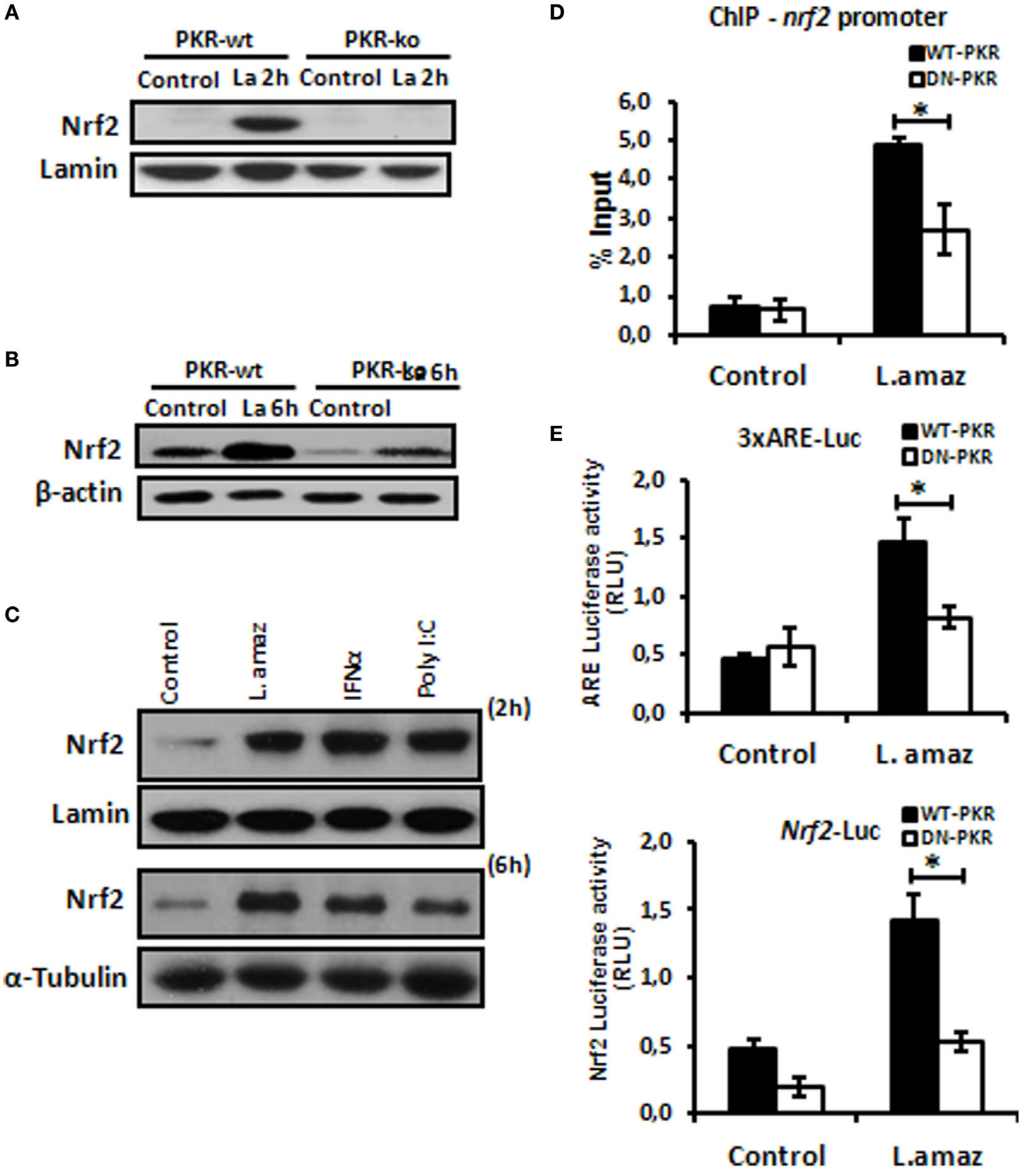
*Leishmania amazonensis* induced nuclear factor erythroid 2-related factor 2 (Nrf2) expression and nuclear translocation in a protein kinase R (PKR)-dependent manner. Peritoneal macrophages from wild-type or PKR-ko 129/sv mice were infected with stationary promastigotes forms of *L. amazonensis* for 2 h **(A)** or 6 h **(B)**. Western-blot was carried out for nuclear or total protein extract, respectively, and then assay was performed using Nrf2 antibody. **(C)** THP-1 cells were infected with *Leishmania amazonensis* or treated with IFN-α or PolyI:C for 2 h for nuclear extract or 6 h for total protein extract, before western-blot analysis with Nrf2 antibody. **(D)** RAW-WT-PKR and RAW-DN-PKR cells were infected with stationary promastigotes forms of *L. amazonensis* for 4 h and then submitted to chromatin immunoprecipitation assay (ChIP) using Nrf2 ChIP-antibody. **(E)** RAW 264.7 cells were transiently transfected with p3xARE- or pNrf2-promoter-luciferase reporter plasmids constructs and infected with *L. amazonensis* 24 h post-transfection. Whole-cell lysates were analyzed for luciferase activity 24 h later. Results are representative of three independent experiments. *p < 0.05.

The oxidative stress response plays a determinant role in the control of intracellular pathogens such as *Leishmania* (32). *L. amazonensis* dampens some macrophage functions, including the induction of oxidative stress (33, 34). Importantly, Nrf2 activation may promote infection tolerance, thus favoring pathogen survival. We sought to investigate whether *L. amazonensis* would induce Nrf2 *via* PKR. Figure 1A and Figure S1A in Supplementary Material shows that Nrf2 translocated to the nuclei of macrophages during the initial phase of interaction with the parasite. Importantly, Nrf2 translocation was not observed in infected pkr-ko and DN-PKR macrophages, respectively. Nrf2 levels were augmented in 6 h of infection and were induced by PKR signaling (Figure 1B; Figure S1B in Supplementary Material). The main target of PKR, eIf2α, is also not activated by phosphorylation in PKR-deficient cells (Figures S1C,D in Supplementary Material). PKR activation by inducers, such as PolyI:C or IFN-I added to macrophages also induced Nrf2 translocation and expression (Figure 1C). Next, we investigated the binding of Nrf2 to cognate *Nrf2* promoter (Figure 1D). Our data show that Nrf2 only occupied the ARE sequences in infected wild-type macrophages by ChIP. To address whether ARE genes are activated in *Leishmania* infection, we constructed two luciferase reporter plasmids. The 3xARE construct contains the canonical ARE promoter response element, while the other construct contains the *Nrf2* promoter (also spanning an ARE-like element). Figure 1E shows that the 3xARE regulatory sequence drove luciferase expression in infected wild-type macrophages, while luciferase expression was abrogated in DN-PKR cells. Importantly, the *Nrf2* promoter was also induced in infected wild-type macrophages. In summary, our results show that *L. amazonensis* induces Nrf2 in a PKR-dependent manner.

### Nrf2 and PKR Signaling Control SOD1 Gene Expression

**Figure 2:**
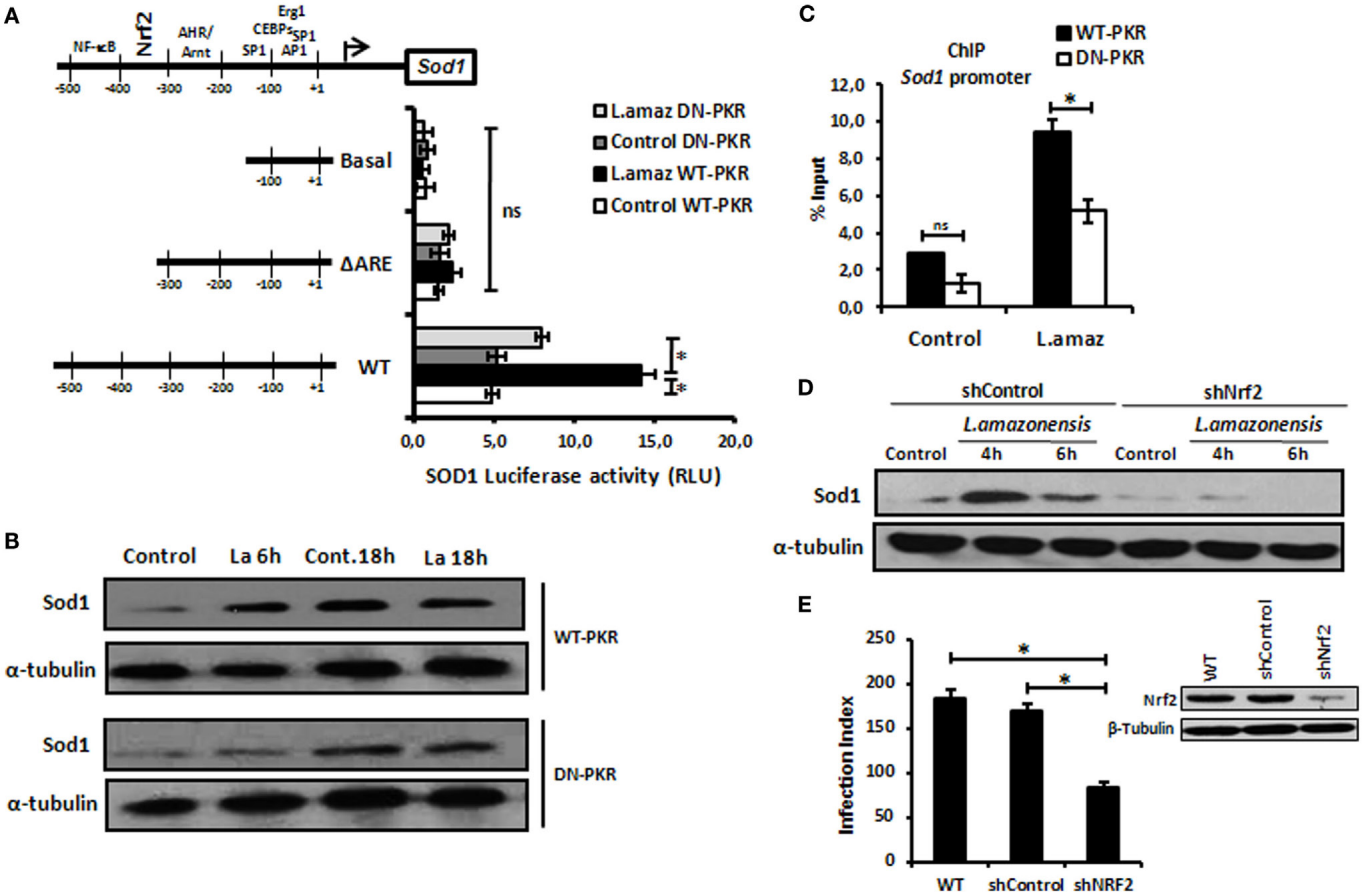
Sod1 regulation through nuclear factor erythroid 2-related factor 2 (Nrf2) and protein kinase R (PKR) signaling. **(A)** RAW 264.7 WT or DN-PKR cells were transiently transfected with Sod1-Luc plasmids and infected with stationary promastigotes of *Leishmania amazonensis* for additional 24 h before luciferase activity assay. **(B)** The same cells were also infected with stationary promastigotes of *L. amazonensis* for 18 h before total protein extract for western-blot analyzes with Sod1 and α-tubulin antibodies. **(C)** RAW 264.7 cells were infected with stationary promastigotes forms of *L. amazonensis* for 4 h and then submitted to chromatin immunoprecipitation assay (ChIP) using Nrf2 ChIP-antibody. shNrf2 or shControl THP-1 cells were infected with stationary promastigotes of *L. amazonensis*, **(D)** Sod1 protein expression was analyzed, and **(E)** infection index was evaluated. Results are representative of three independent experiments. *p < 0.05.

Recent reports have demonstrated that *L. amazonensis* activates the classical antiviral response mediated by PKR, leading to Sod1 expression, favoring parasite growth in infected macrophages (35, 36). To address the role of Nrf2 on Sod1 expression in infected macrophages, we cloned the *Sod1* promoter and deleted the regulatory regions in the Luciferase vector (pGL2) (Figure 2A). The *Sod1* promoter was induced in wild-type infected macrophages, while the deletion of the ARE sequence disrupted Luc expression. Accordingly, Sod1 expression, which is controlled by Nrf2, was only increased in wild-type infected macrophages (Figure 2B). Our data show that ARE element on Sod1 promoter was occupied by Nrf2 only in infected wild-type macrophages by ChIP (Figure 2C). We aimed to test the hypothesis that Sod1 dependence of Nrf2 activity, we developed a macrophage shNrf2 knockdown cell line. In only wild-type infected macrophages, the parasites induce Sod1 expression (Figure 2D). The quantification of infection index show a decrease on proliferation of *Leishmania* in Nrf2 knockdown cells (Figure 2E). These data support the link between Sod1 and two major signaling pathways represent by Nrf2 and PKR.

### Akt1 Controls Nrf2 Induction in Infected Macrophages

**Figure 3:**
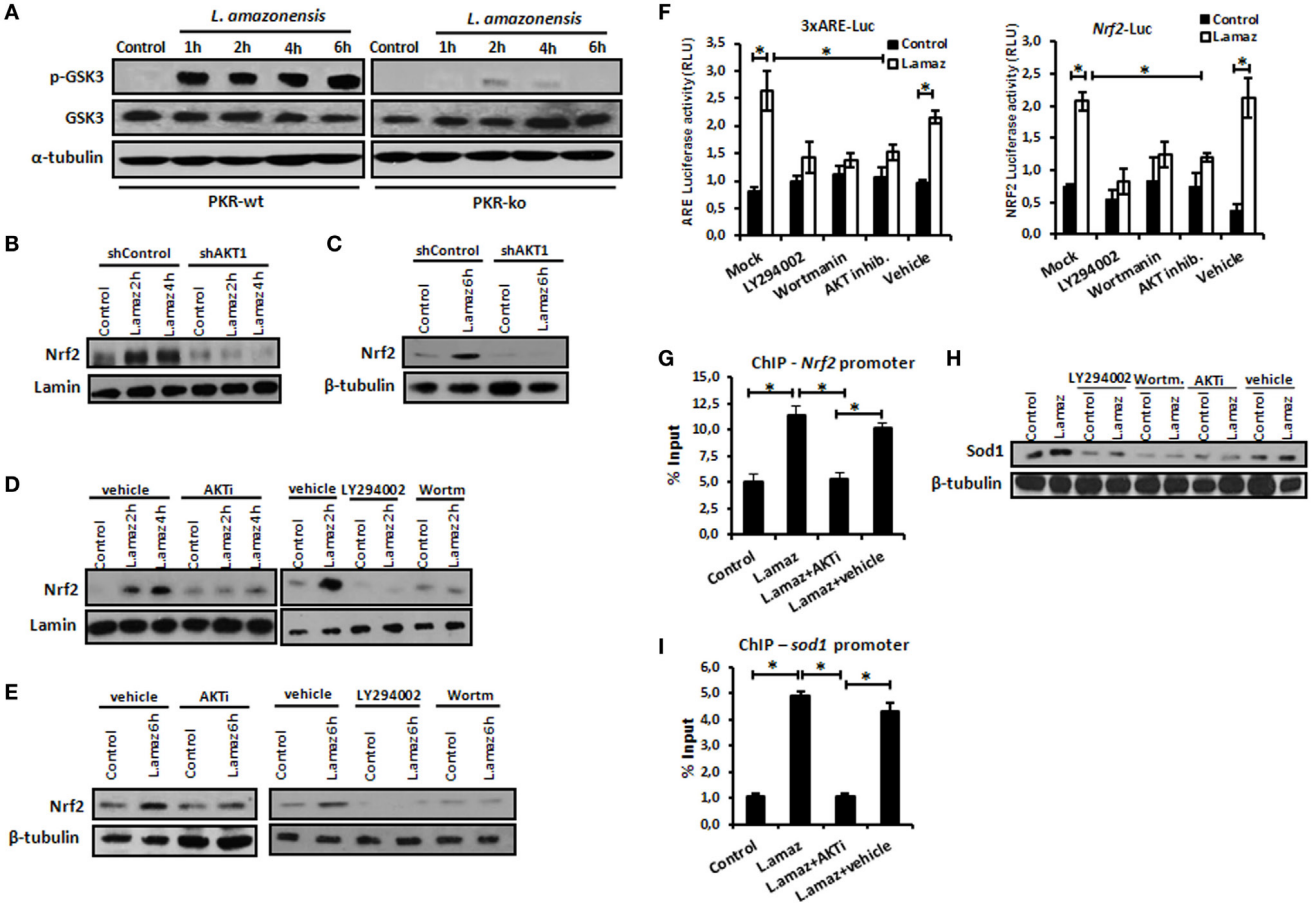
Protein kinase R (PKR)-dependent PIK3/Akt signaling activation controls positively the nuclear factor erythroid 2-related factor 2 (Nrf2) functions in *Leishmania*-infected macrophages. **(A)** Peritoneal macrophages from wild-type or PKR-ko 129/sv mice were infected with stationary promastigotes forms of *Leishmania amazonensis* at indicated times. Western-blot was carried out for total protein extract with anti-phospho-GSK3 and anti-GSK3. THP-1 cells stably knocked-down for Akt1 expression **(B,C)** and treated with PI3K/Akt inhibitors (LY294002, Wortmannin and Akt-inhibitor-VIII) **(D,E)** were infected with stationary promastigotes forms of *Leishmania amazonensis* at indicate times. Nuclear and total protein extracts were analyzed using Nrf2 antibody. **(F)** THP-1 cells were transiently transfected with p3xARE- or pNrf2-promoter Luciferase reporter plasmids. Twenty-four hours post-transfection, cells were differentiated into macrophages with phorbol-12 myristate-13 acetate (PMA) treatment for 6 days. The cells were infected with stationary promastigotes forms of *L. amazonensis* and/or treated with PI3K/Akt inhibitors for additional 24 h. Whole-cell lysates were analyzed for luciferase activity 24 h later. THP-1 cells were infected with stationary promastigotes forms of *L. amazonensis* and/or treated with Akt-inhibitor-VIII for 4 h and then submitted for chromatin immunoprecipitation assay (ChIP) using Nrf2 ChIP-antibody and primers for *Nrf2* **(G)** and *Sod1* **(I)** promoters. **(H)** Western-blot for total protein extract analyses with Sod1 antibody was performed at same conditions of infection and treatment. Results are representative of three independent experiments. *p < 0.05.

Nrf2 activation is controlled at different levels, including indirect phosphorylation by Akt1 (11). Because *L. amazonensis* promotes Akt1 activation (12), we aimed to investigate its role in Nrf2 induction. Initially, we examined whether the induction of Akt1 by *L. amazonensis* relied on PKR expression. Figure 3A shows that GSK3 phosphorylation due to Akt1 depended on PKR. The phosphorylation of Akt depends on PKR during *Leishmania* infection (Figures S3A,B in Supplementary Material). Of note, Nrf2 induction required Akt signaling, as shown in infected shAkt1 macrophages (Figures 3B,C). In macrophages treated with pharmacological inhibitors of Akt1/2 and PI3K (Figures 3D,E), we also observed the same pattern of Nrf2 repression in nucleus translocation and protein expression. As predicted, ARE element, *Nrf2* Luciferase and *Nrf2* promoter occupancy in ChIP assay were induced by *L. amazonensis* infection in an Akt1-dependent manner (Figures 3F,G). Likewise, Sod1 expression followed the same PI3K/Akt1 dependence pattern. ChIP assays corroborated these findings, where the occupancy of ARE in the *Sod1* promoter by Nrf2 depended on Akt1 (Figure 3H).

### Nrf2 Knockdown Promotes Oxidative Stress and Impairs Parasite Survival in Macrophages

**Figure 4:**
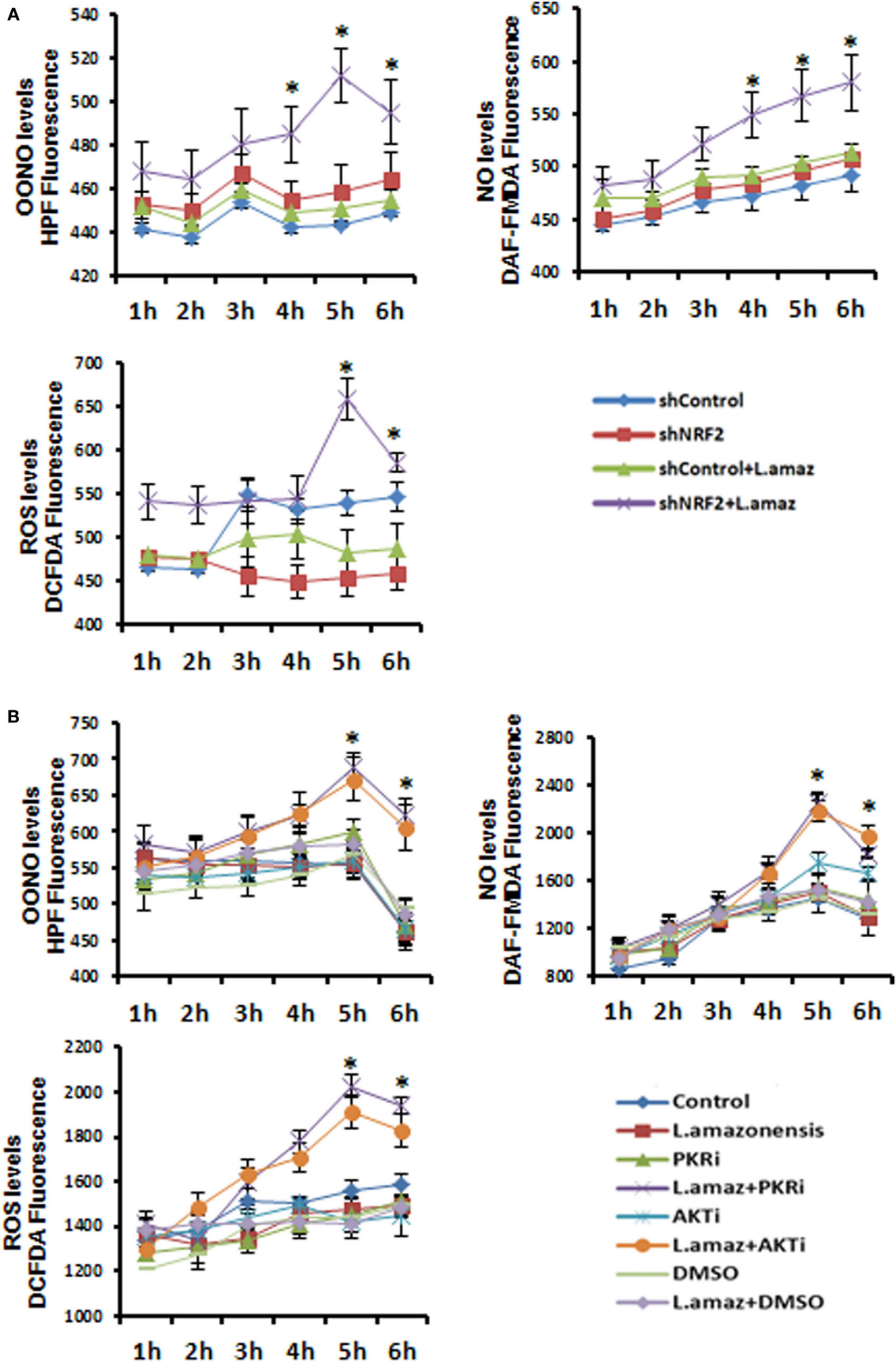

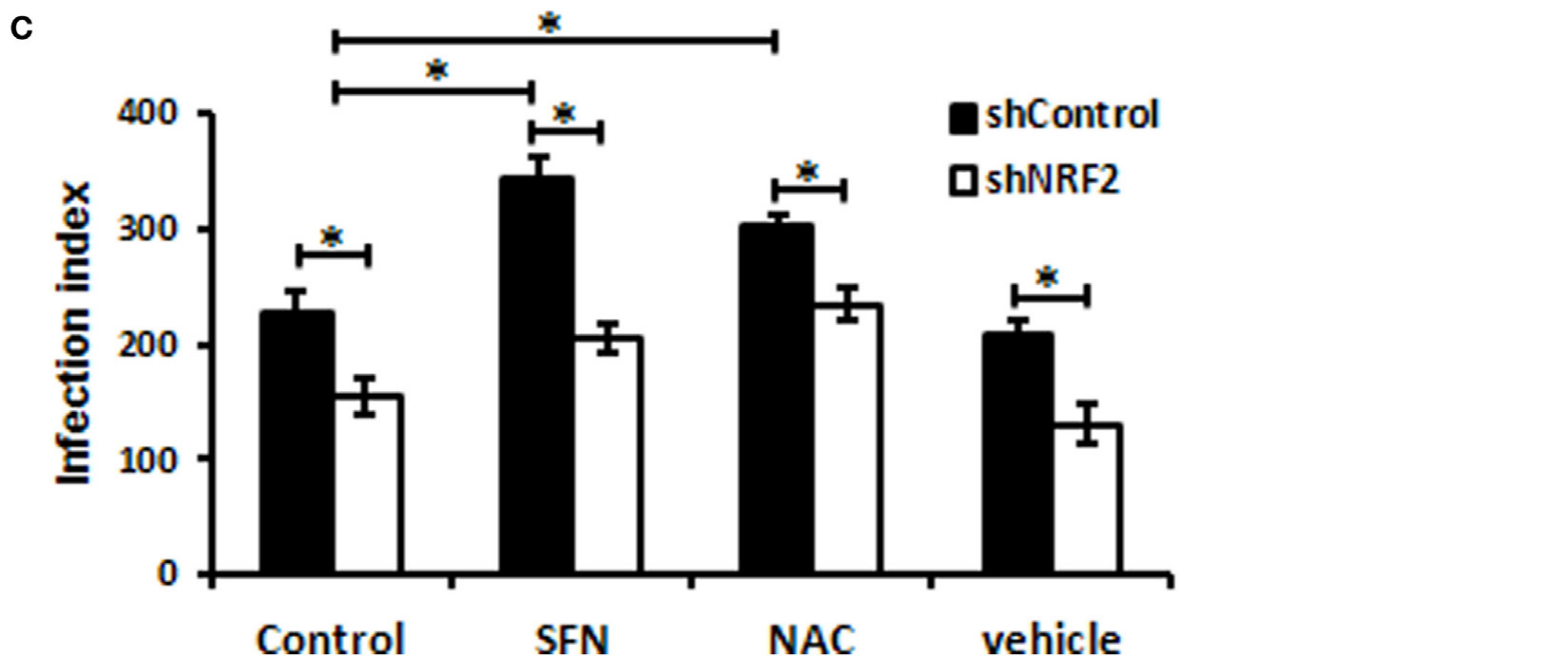
The reactive oxygen species (ROS) were enhanced upon *Leishmania amazonensis* infection in nuclear factor erythroid 2-related factor 2 (NRF2)/protein kinase R (PKR)/Akt-deficient macrophages. **(A)** shNrf2 or shControl and **(B)** wild-type THP-1 cells treated with PKR-inhibitor or Akt-inhibitor-VIII were infected with stationary promastigotes forms of *L. amazonensis* at indicated times together with probes for quantifying peroxynitrite (OONO), nitric oxide (NO), and ROS, and then analyzed as described in material and methods. **(C)** THP-1 transiently knocked-down for Nrf2 expression or shControl cells were infected with stationary promastigotes forms of *L. amazonensis* for 24 h and treated for additional 24 h with sulforaphane (SFN) or NAC (N-acetylcysteine) before the analysis of infection index. The asterisk means the statistic significant differences between the groups. Results are representative of three independent experiments. *p < 0.05.

We aimed to test the hypothesis that Nrf2 knockdown would favor oxidative stress, leading to the reduction of the parasite load in macrophages. We measured the production of OONO, NO, and ROS as components of the oxidative stress pathway in Nrf2-knockdown infected macrophages (Figure 4A). As expected, the production of ROS and the formation of OONO and NO were enhanced in infected Nrf2-knockdown macrophages. Figure 4B shows that PKR or Akt1 inhibition leads to a similar oxidative stress profile upon infection. Silencing of Nrf2 decreased the infection index, whereas the parasite load was rescued when infected Nrf2-knockdown macrophages were treated with the antioxidant NAC compound (Figure 4C). Notably, the Nrf2 inducer sulforaphane augmented the infection index.

### *Leishmania* Down-Regulates the Nrf2 Negative Regulator Keap1 and Induces Autophagy

**Figure 5:**
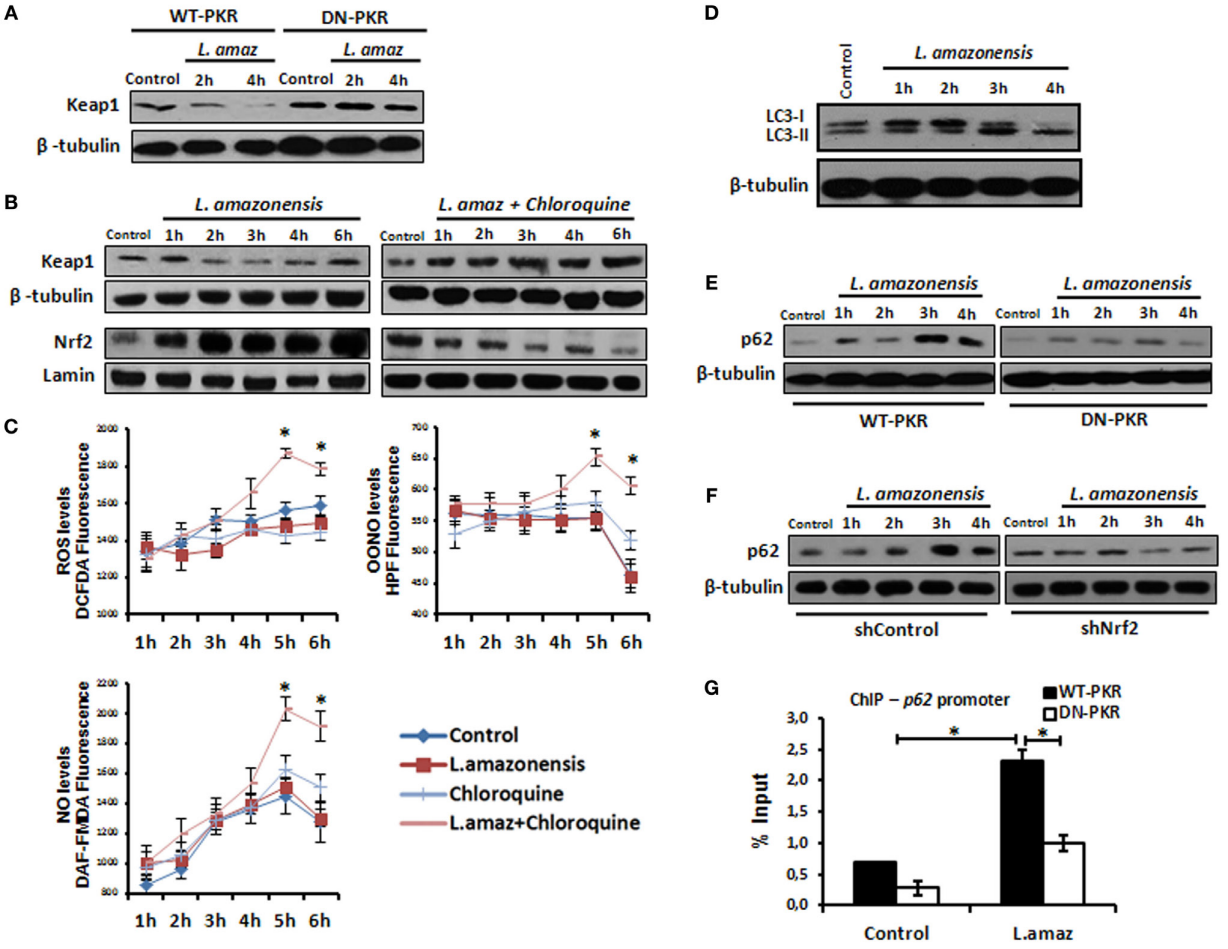
The nuclear factor erythroid 2-related factor 2 (Nrf2)-inhibitor Kelch-like ECH-associated protein 1 (Keap1) is modulated negatively through protein kinase R (PKR) signaling and p62 autophagy-dependent manner in *Leishmania* infection. **(A)** RAW-WT-PKR and RAW-DN-PKR cells were infected with stationary promastigotes forms of *Leishmania amazonensis* for 2 or 4 h and then western-blot assay were performed with total protein extract using Keap1 antibody. **(B)** THP-1 cells were infected with stationary promastigotes forms of *L. amazonensis* at indicate times and/or treated with chloroquine and the total or nuclear protein extracts were analyzed using Keap1 and Nrf2 antibodies. **(C)** THP-1 cells treated with chloroquine were infected with stationary promastigotes forms of *L. amazonensis* at indicated times together with probes for quantifying OONO, NO, and ROS. **(D)** THP-1 cells were infected with stationary promastigotes forms of *L. amazonensis* and western-blot for LC3-I/II protein was performed. RAW-WT-PKR and RAW-DN-PKR cells **(E)**, and shNrf2 or shControl THP-1 cells **(F)** were infected with stationary promastigotes forms of *L. amazonensis* and then the total protein extract was analyzed by western-blot assay with p62 antibody. **(G)** RAW-WT-PKR and RAW-DN-PKR were infected with stationary promastigotes forms of *L. amazonensis* for 4 h and then submitted for ChIP assay using Nrf2 ChIP-antibody and primers for *p62* promoter. Results are representative of three independent experiments. *p < 0.05.

Nuclear factor erythroid 2-related factor 2 is sequestered in the cytosol by a homodimer of Keap1, which limits its nuclear translocation. Nrf2 associated with Keap1 is directed to proteasomal degradation by Cul3-mediated poly-ubiquitination (37). However, Keap1 is degraded through p62-mediated autophagy, releasing Nrf2 into the nucleus (38). Given that *Leishmania* induces autophagy in infected macrophages (39), we sought to investigate the levels of Keap1 in *Leishmania* infection. Figure 5A shows the prompt decrease in Keap1 levels in infected macrophages and demonstrates that PKR inactivation prevented Keap1 degradation. Moreover, Keap1 reduction was prevented by chloroquine, an autophagy inhibitor (Figure 5B). Given that Nrf2 released *via* Keap1 degradation promotes the antioxidant response, we addressed whether the inhibition of autophagy would increase the oxidative stress of infected macrophages. As observed in Figure 5C, the levels of ROS, OONO, and NO increased in infected cells treated with chloroquine. We also confirmed that *L. amazonensis* triggers LC3-I conversion to LC3-II, a marker of autophagy (Figure 5D). Given that the formation of the LC3-p62-Keap1 ternary complex on the autophagosome membrane directs Keap1 to degradation (40), we investigated the induction of p62 in the infection. We show that p62 was induced in infected macrophages, and this effect relied on PKR and Akt1 (Figure 5E and Figures S5A in Supplementary Material, respectively). Moreover, the ablation of Nrf2 expression prevented p62 induction due to infection (Figure 5F). Given that our data indicate that PKR and Akt control the induction of Nrf2, we tested the occupancy of the *p62* promoter by Nrf2 in the context of infection. Our data show that *Leishmania* promoted Nrf2 occupancy, and the inhibition of either PKR or Akt signaling prevented this effect (Figure 5G and Figure S5B in Supplementary Material, respectively).

### Nrf2 Protein Levels Are Elevated in Human CL, and *L. braziliensis* also Induces Nrf2 *In Vitro*

**Figure 6:**
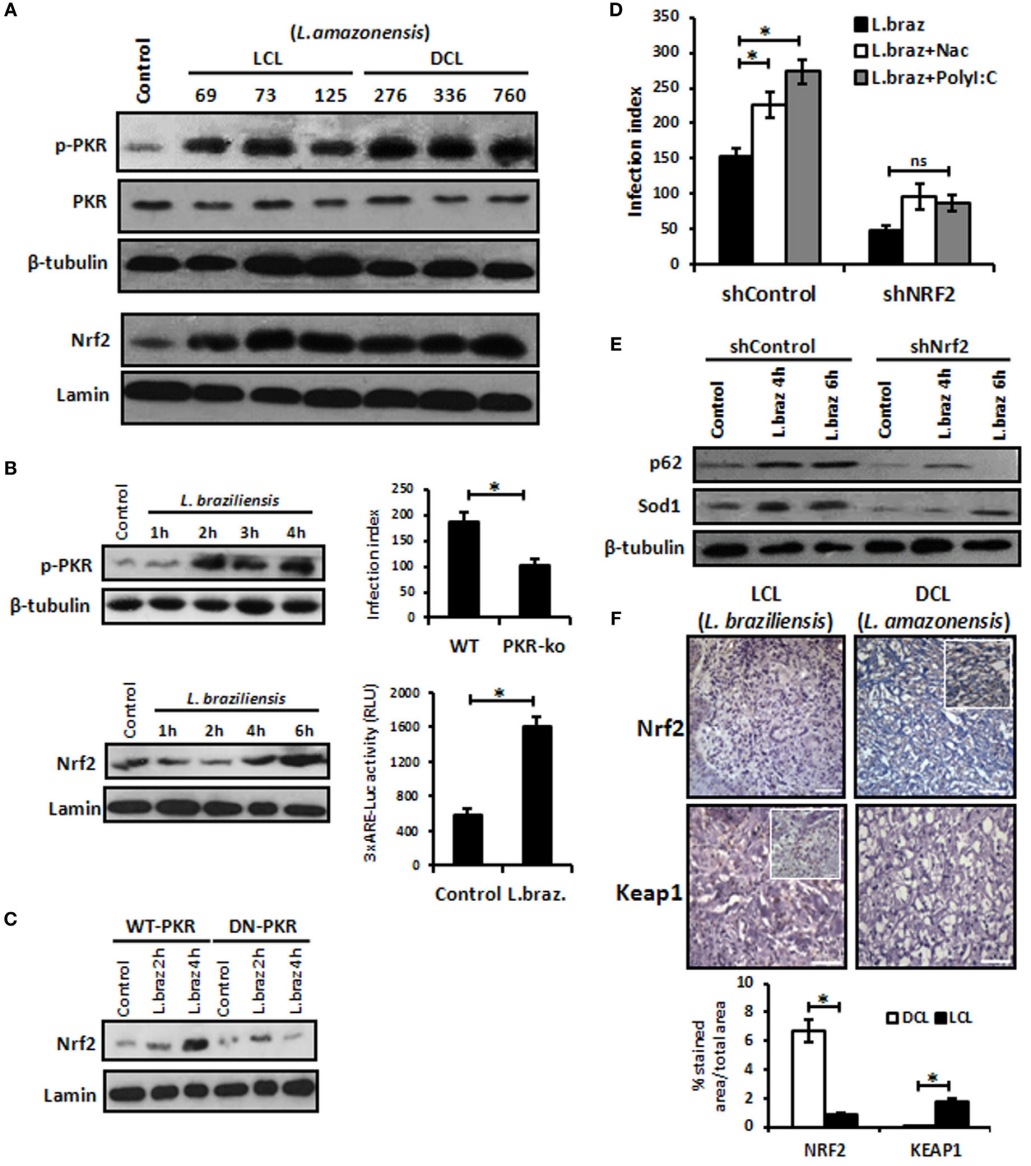
*Leishmania braziliensis* and different strains of *Leishmania amazonensis* infections also modulated positively the protein kinase R (PKR)/nuclear factor erythroid 2-related factor 2 (Nrf2) axis pathway. **(A)** *L. amazonensis* strains from LCL or diffuse cutaneous leishmaniasis (DCL) patients were used to infect THP-1 cells. Total or nuclear protein extracts were processed and then analyzed by western-blot with phospho-PKR and Nrf2 antibodies, respectively. **(B)** THP-1 cells were infected with stationary promastigotes forms of *L. braziliensis* at indicate times and then performed for western-blot with phospho-PKR and Nrf2 antibodies, and 3xARE-promoter Luciferase assays. Peritoneal macrophages of wild-type or PKR-ko mice were infected with *L. braziliensis* and infection index assays were then analyzed. **(C)** RAW-WT-PKR and RAW-DN-PKR cells were infected with *L. braziliensis* and western-blot with anti-Nrf2 was then analyzed. **(D)** shNrf2 or shControl THP-1 cells were infected with stationary promastigotes forms of *L. amazonensis* for 24 h before treatment with NAC or polyI:C for additional 24 h. After this time, the cells were fixed and the infection index was evaluated. **(E)** THP-1 transiently knocked-down for Nrf2 expression or shControl cells were infected with stationary promastigotes forms of *L. amazonensis* for 4 or 6 h before total protein extract for western-blot analyzes with p62 and Sod1 antibodies. **(F)** Histological sections from biopsies obtained from lesions of patients with DCL (*n* = 4) or with LCL (*n* = 5) were submitted to immunohistochemical reaction with primary antibodies against Nrf2 or Keap1 as previously described. All sections were counterstained with hematoxylin. Digital images (400× magnification) were captured using a Nikon E600 microscope and an Olympus Q-Color 1 digital camera with the Image Pro Plus program. Bars represent 10 µm. Positive cell density was obtained. Graph represents the analysis of reactive positive cells for Nrf2 and Keap1 compared with isotype controls as percentage of positive stained area per total tissue area. Results are representative of three independent experiments. *p < 0.05.

We aimed to address whether other *L. amazonensis* strains isolated from patients with localized cutaneous lesions (LCL) or DCL would induce Nrf2 nuclear translocation and the activation of PKR. Figure 6A shows that all distinct strains of*L. amazonensis* activated PKR and Nrf2. Given that most of the cases of human CL in Brazil are caused by *L. braziliensis*, we decided to address whether this species would induce PKR and Nrf2. Figure 6B shows that *L. braziliensis* activated PKR and Nrf2. Nrf2 activation depended on PKR function (Figure 6C). Moreover, the expression of the Nrf2 target genes p62 and Sod1 was reduced in Nrf2-silenced *L. braziliensis*-infected macrophages (Figure 6E), and the growth of amastigotes was impaired in Nrf2-knockdown macrophages (Figure 6D). These results prompted us to investigate the levels of Nrf2 and the negative regulator Keap1 in clinical samples from LCL or DCL patients. Figure 6F shows the marked expression of Nrf2 in DCL samples compared to LCL tissues. Accordingly, Keap1 expression was enriched in LCL samples. Altogether, the data show that Nrf2 induction is triggered by distinct species and strains of *L. amazonensis*, and high levels of Nrf2 are found in patients with DCL, a severe clinical condition that presents with a high number of parasites and poor prognosis (41).

### Transcriptomic Analysis Reveals a Pivotal Role of Nrf2 Signaling in CL Patient Samples

Next, we tested for transcription factor enrichment among the 413 genes composing the systemic LCL disease signature. Only five transcription factor motifs were significantly enriched among the promoters of the 413 genes of the LCL disease signature. After the E4F1 motif, the Nrf2 binding site was the second-most significantly represented, being present in 15 of the 413 genes composing the LCL disease signature (Table 1). Among those, p62 (Sqstm1), in bold, was confirmed, in agreement with our *in vitro* data. We herein present the first disease signature of LCL using a systems biology analysis of the PBMC transcriptome of LCL patients (*n* = 18) vs. healthy controls (*n* = 12). Using Affymetrix microarrays (HuGene 1.0), we found that Nrf2 was significantly overexpressed in patient PBMCs vs. controls (1.8-fold, uncorrected *p* = 0.0002, *p* = 0.033 using the Benjamini–Hochberg correction for genome-wide testing). The top 50 upregulated genes in patients vs. controls are shown in Table S1 in Supplementary Material. Next, we used IPA to determine which biological pathways and molecular networks were enriched among the LCL disease signature. As shown in Table S2 in Supplementary Material, three antioxidant pathways, i.e., the thioredoxin pathway, the antioxidant action of Vitamin C and the Nrf2 pathway, were significantly enriched in the LCL disease signature.

**Table 1:** Gene promoters in LCL disease signature are enriched for nuclear factor erythroid 2-related factor 2 transcription factor binding sites.

Index	Gene symbol	Gene name	Entrez gene
1	TXNRD1	Thioredoxin reductase 1	7296
2	TFAP4	Transcription factor AP-4	7023
3	**SQSTM1**	**Sequestosome 1**	**8878**
4	RB1CC1	RB1-inducible coiled-coil 1	9821
5	CDH23	Cadherin-related 23	64072
6	SLC16A6	Solute carrier family 16, member 6	9120
7	KBTBD8	Kelch repeat and BTB (POZ) domain containing 8	84541
8	FBXO30	F-box protein 30	84085
9	ATP1B1	ATPase, Na+/K+ transporting, beta 1 polypeptide	481
10	PRDM1	PR domain containing 1, with ZNF domain	639
11	MAST2	Microtubule-associated serine/threonine kinase 2	23139
12	CLC	Charcot–Leyden crystal protein	1178
13	SYTL1	Synaptotagmin-like 1	84958
14	SFXN5	Sideroflexin 5	94097
15	TMEM57	Transmembrane protein 57	55219

### Nrf2 Transcriptome-Wide Correlations Confirm the Links between IFN-I/PKR, ARE, PIK3, and Autophagy Signaling Pathways *In Situ*

**Figure 7:**
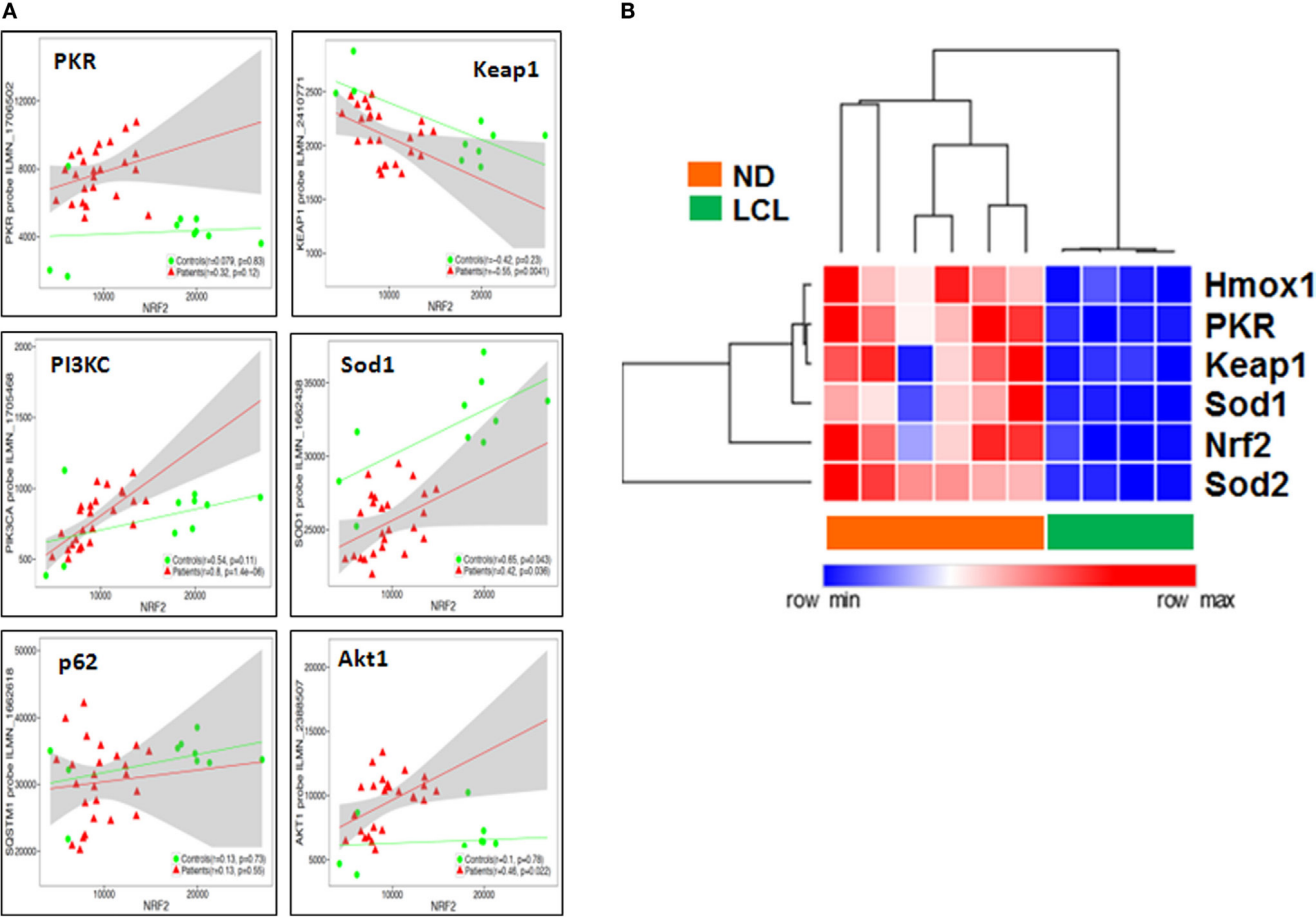
Nuclear factor erythroid 2-related factor 2 (Nrf2) transcriptome-wide correlations confirm the links between IFN/protein kinase R (PKR), antioxidant response element (ARE), PIK3, and autophagy signaling pathways *in situ*. **(A)** Transcriptome-wide correlation analysis was applied to Nrf2 transcript levels in microarray data (Illumina) obtained from skin biopsies from LCL patients (*n* = 20, red) and healthy controls (*n* = 10, green). Spearman correlation coefficient and *p*-values are shown for individual transcripts, 95% confidence intervals are shown in gray for patients only. **(B)** Validation of increased PKR/Nrf2 signaling pathway members by digital mRNA quantification. Heat map and hierarchical cluster analysis (Eucledian distance) of selected genes quantified by nCounter digital transcriptomics (Nanostring) in skin biopsies from normal donors (ND, *n* = 4) and LCL patients (*n* = 6), normalized according to PTPRC (CD45) expression levels, to account for differences in tissue leukocyte infiltration.

Then, we performed a transcriptome-wide correlation analysis to further investigate whether the molecular links we described at the protein level *in vitro* might be confirmed at the transcriptional level *ex vivo*. The expression of a large number of genes was significantly correlated to Nrf2 transcript levels, even following stringent Benjamini–Hochberg correction for multiple testing. Among those, *PKR, PIK3C, Sod1*, and *p62* (*SQSTM1*) transcripts were positively correlated, whereas *Keap1* was negatively correlated, to *Nrf2* transcript levels, with minor differences between LCL patients and controls (Figure 7A), thus confirming our protein data of *Nrf2* regulation, both upstream or downstream. To validate these microarray results, we performed a targeted analysis of key genes in the Nrf2/PKR crosstalk using nCounter digital transcriptomic quantification in LCL (*n* = 6) as well as healthy skin biopsies (*n* = 4). As shown in Figure 7B, unsupervised hierarchical clustering of *in situ* transcriptomes revealed two major clusters, which coincided with either LCL patients or normal donors (ND). Thus, LCL skin biopsies could be discriminated from healthy skin by differential expression of only six transcripts (*Nrf2, PKR, Sod1, Sod2, Keap1*, and *Hmox1*).

## Discussion

The oxidative burst in infected cells is a key microbicide mechanism exhibited by macrophages. However, *Leishmania* parasites present a repertoire of adaptive mechanisms to cope with the altered redox state of infected macrophages by expressing antioxidant enzymes or interfering with macrophage signaling pathways (42). A growing number of reports indicate that PKR modulates infections caused by intracellular pathogens (43). Notably, increased levels of Sod1 are expressed in macrophages infected by *L. amazonensis* due to PKR activation (35). Given that the transcription factor Nrf2 is the main regulator of Sod1 expression (44) among other genes involved in the anti-oxidative response, we studied the regulation of Nrf2 in the context of *Leishmania* infection and tested the hypothesis that PKR is actually a positive regulator of the ARE *via* Nrf2.

Our results demonstrated that Nrf2 activation depended on PKR signaling. Simple treatment with PKR inducers such as IFN-α and PolyI:C increased the expression and nuclear translocation of Nrf2, demonstrating that the mechanisms of Nrf2 activation through PKR pathway signaling are not exclusively due to *Leishmania* infection. Remarkably, PKR activation induces a significant increase in Nrf2 expression. PKR phosphorylates eIF2-α, which reduces protein synthesis while upregulating the expression of some genes such as ATF-4. The 5′untranslated region of Nrf2 mRNA presents an internal ribosomal entry site (IRES), allowing enhanced Nrf2 translation in eIF2-α-mediated protein translation (45, 46).

The control of Nrf2 activation requires different post-translational modifications as well as its repression and subsequent degradation via the proteasome (8–11, 47). The non-canonical PI3K/Akt signaling pathway has been linked to the activation of Nrf2 in a number of models. GSK3-mediated inhibitory phosphorylation induces Nrf2 by inhibiting the phosphorylation signal and sequential ubiquitination in the Neh6-Nrf2 domain, allowing its stability and activity (13). In *Leishmania* infection, PI3K and Akt inhibition reduced the expression of Nrf2 and Sod1 (Figure 3). Our results led us to conclude that PI3K/Akt activation as a result of *Leishmania* infection is a positive Nrf2 regulator in host cells.

The rise of ROS seems to be a key regulator of infection by intracellular pathogens (48), and the co-evolution of host cells and parasites results in a shared pattern of subversion in the production of these radicals. For example, *Trypanosoma cruzi* infection in THP-1 cells requires a level of oxidative stress for successful parasitism, given that the overexpression of Nrf2 reduces parasitism (49). Our data from *in vitro* Nrf2-knockdown macrophages revealed the spontaneous increase of oxidative stress, measured through the levels of ROS, NO, and OONO. The same change in phenotype occurred when PKR and Akt were inhibited, probably due to the reduction of Sod1 and other targets. However, the infection index increased when the cells were treated with SFN and NAC. Our data suggest that Nrf2 activation induces Sod1, thus counteracting the oxidative boost in the cell milieu in infected macrophages.

Several reports (50) have highlighted the close relationship between oxidative stress and the autophagy process. The autophagy pathway plays an important role in resistance to various infections, although it could be subverted, thus favoring some infections (51). It is conceivable that autophagy induced by *L. amazonensis* may be controlled by PKR, as revealed in other models, thus regulating Nrf2 levels. Accordingly, some studies have shown the importance of oxidative stress sensing in autophagy (15, 16) and have demonstrated that the degradation of Keap1 *via* autophagy allows cellular redox homeostasis in liver cells. Our data showed that infected macrophages display an increase in LC3-I to LC3-II conversion, thus corroborating the importance of autophagy through this marker during infection.

Kelch-like ECH-associated protein 1 is a negative regulator of Nrf2, and in the context of infection by *Leishmania*, we demonstrated that Keap1 is regulated after 18 h of infection in a PKR-independent manner (data not included). However, Keap1 stability is decreased in a PKR-dependent manner between 2 and 4 h of infection. When autophagy was inhibited by chloroquine, we noted a cytoplasmic accumulation of Nrf2 and stabilization of Keap1 levels, which was accompanied by high levels of oxidative stress.

Several studies have shown the involvement of the p62 (Sqstm1) protein as a central regulator between Keap1 and Nrf2. Oxidative stress decreases when cells overexpressing p62 bind to this inhibitory protein, leading to autophagosome formation (19, 20). Other studies have shown that Nrf2 positively regulates the expression of p62 (17). Importantly, TLR2 activation culminates in M2 polarization of macrophages (MOX macrophages), which leads to NF-κB-p65 degradation through p62 and lysosomes, characterized by selective autophagy (52). M2 macrophages exhibit antioxidant properties, as judged by the expression of Cox2, IL1β, HO-1, VEGF, and Nrf2 (53). Considering the cascade of signals, our data support the notion that the PKR–PI3K/Akt↔Nrf2 axis regulates p62 gene expression in *Leishmania* infection and passively triggers the autophagy pathway that culminates in Keap1 degradation, activating Nrf2 and resulting in oxidative cellular homeostasis.

Patients with localized cutaneous lesion (LCL) exhibit predominant expression of iNOS, IL-1β, IL-6, MCP-1, TNF-α, and IFN-γ, while anergic diffuse cutaneous leishmaniasis (ADCL) lesions are characterized by the presence of IL-4, IL-5, IL-10, and MIP-1α and the low expression of iNOS (54, 55). Our *in vitro* data showed that *L. braziliensis*, the prominent causative agent of LCL, also induces Nrf2 in a PKR-dependent fashion. This observation underlines the importance of this signaling pathway in other *Leishmania* species besides *L. amazonensis*. However, the immunohistochemistry analysis of LCL vs. DCL lesions revealed a strong Nrf2 reaction in the latter group, while the Keap1 signal was predominant in the former clinical samples. These results indicate that Nrf2 activation may contribute to the poor oxidative response and, consequently, the high parasite burden in DCL patients.

The data obtained in this study confirm and extend our previous finding of an IFN-I/Sod1 axis, linked to increased parasite burden (56) and therapeutic failure in both localized cutaneous leishmaniasis and DCL (31). This study now reveals that this IFN-I/Sod1 link is critically mediated by Nrf2/ARE signaling. Our genome-wide study revealed Nrf2 as a master regulator of the *in situ* (skin biopsies) transcriptome (Figure 7), both in health and disease, which is in agreement with its central role in proteostasis and ancient molecular networks, conserved in evolution from Drosophila to man (57). There is a negative correlation with the Keap1 transcript skin biopsies, which was corroborated by our findings at the protein level in both LCL and DCL skin biopsies (Figure 6F). These results point to a possible compartmentalization of the pathogen-driven immune response between tissues in CL, where cutaneous ulcers in LCL are exposed to a complex microbiome, which strongly influences the local immune response, in addition to *Leishmania* antigens (58).

**Figure 8:**
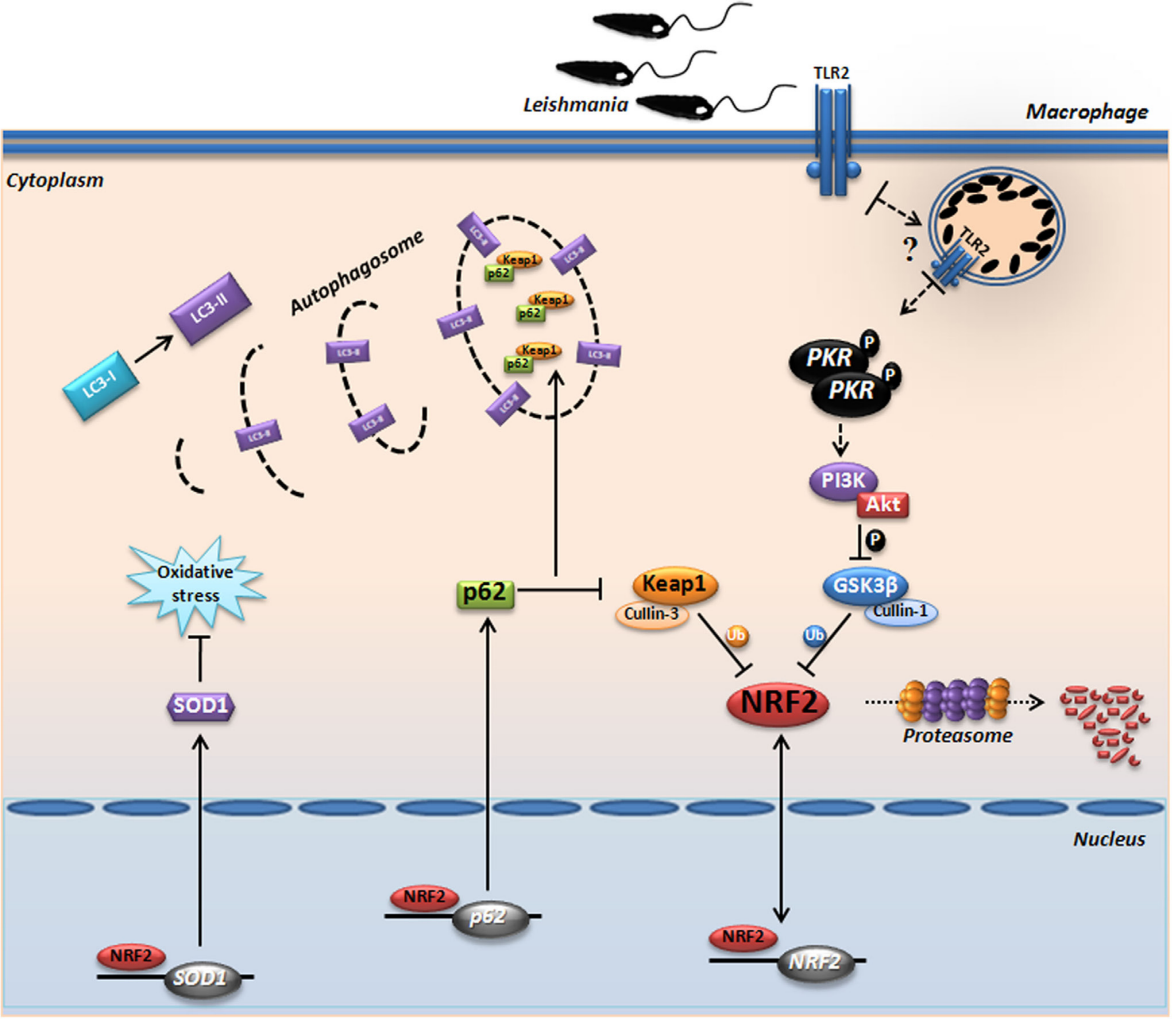
Proposed model for the protein kinase R (PKR)-dependent nuclear factor erythroid 2-related factor 2 (Nrf2) activation in *Leishmania* infection. Internalized parasite signals through the endosomal compartment *via* TLR2 and induce activation of PKR by dimerization and subsequent autophosphorylation. Subsequently, we found that GSK3 phosphorylation is dependent of PKR signaling, allowing that not occur inhibition of Nrf2 through Neh6 inhibitory domain. This activation of Nrf2 is also dependent of Keap1 inhibition through of autophagic and PKR pathways. These mechanisms induce nuclear translocation Nrf2, increasing the gene expression of *Sod1, Nrf2*, and *p62*. The sequestosome-1 (p62) could be recruiting, together with processed LC3-II and Keap1 for autophagic vacuoles, allowing greater Nrf2 activation and inhibition of oxidative stress through antioxidant enzymes.

Due to its strong pleiotropic effects and its essential function in normal homeostasis, Nrf2 itself is not yet a target of choice for therapeutic intervention in LCL. However, this study reiterates our previous suggestion that downstream targets of Nrf2, such as Sod1, represent excellent therapeutic targets in LCL. Previous works from our and other groups (59–62) have shown that the Sod1 inhibitor DETC or its precursor molecule, disulfiram, are plausible therapeutic alternatives that have been used extensively in humans for decades with an excellent safety profile. In conclusion, we demonstrate for the first time the prominent role of Nrf2 and the PKR↔PI3K/Akt↔p62/autophagy axis in human and experimental leishmaniasis (Figure 7B). Collectively, our data propose a signaling-based scenario that may reveal a pivotal molecular basis for CL pathogenesis as well as its therapeutic potential. A schematic model based in our results is depicted in Figure 8.

## Ethics Statement

Written informed consent was obtained from all participants or legal guardians, and all of the data analyzed were anonymized. The project was approved by the Institutional Review Board of Centro de Pesquisas Gonçalo Moniz, FIOCRUZ–BA (license number 136/2007) and complies with the guidelines of the Declaration of Helsinki.

## Author Contributions

AV—designed and performed experiments, analyzed data, and wrote the manuscript; TC-S—performed initial experiments for the study; AS—provided reagents, supervised experiments, and critically evaluated the manuscript; VB, JF-C, RK, AB, TD, JW, and VB—carried out experiments with patient samples, analyzed the results, and provided input for experimental design and interpretation; NF—critically reviewed the manuscript and analyzed data, and UL—directed the study, analyzed the data, and wrote the manuscript.

## Conflict of Interest Statement

The authors declare that they have no conflicts of interest with the contents of this article. The handling editor declared a shared affiliation, though no other collaboration, with several of the authors, AV, TS, KD, and UL.
