# Supplementary material for: Systems Approach Reveals Nuclear Factor Erythroid 2-Related Factor 2/Protein Kinase R Crosstalk in Human Cutaneous Leishmaniasis
Source: Front Immunol. 2017 Sep 15;8:1127. doi: 10.3389/fimmu.2017.01127 (PMC5605755; doi:10.3389/fimmu.2017.01127)
Supplement: Supplementary file 1 [file image_1.pdf]

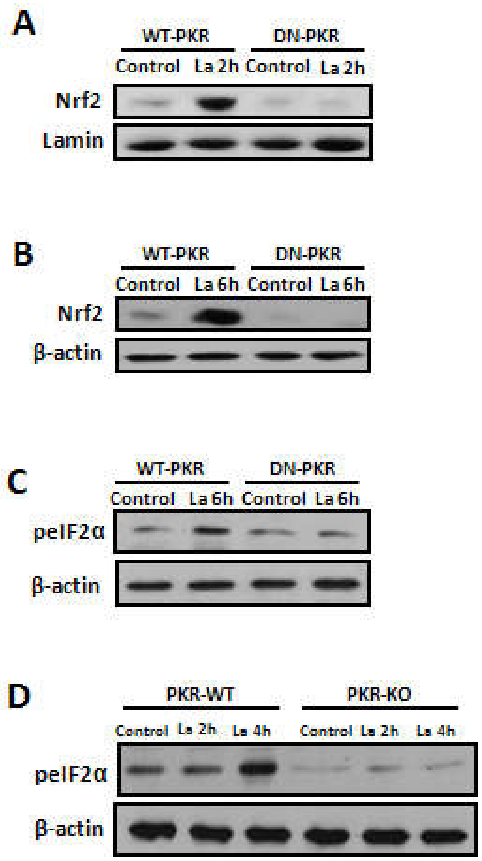

FIGURE S1. PKR signaling modulated Nrf2 activation and eIF2α phosphorylation in a *Leishmania amazonensis* infection.

RAW-WT-PKR or the dominant-negative PKR K296R cells (RAW-DN-PKR) were infected with stationary promastigotes forms of *L. amazonensis* for 2 hours **(A)** or 6 hours **(B)** and western-blot was carried out for nuclear or total extract with anti-Nrf2, respectively. The same cells RAW WT or DN-PKR **(C)** or PKR-WT and PKR-KO peritoneal macrophages **(D)**, were also infected with stationary promastigotes forms of *L. amazonensis* for 6 hours, or 2h-4h, respectively, and then total protein extract was analyzed by western-blot assay using phospho-eIF2α antibody. Results are representative of 3 independent experiments.

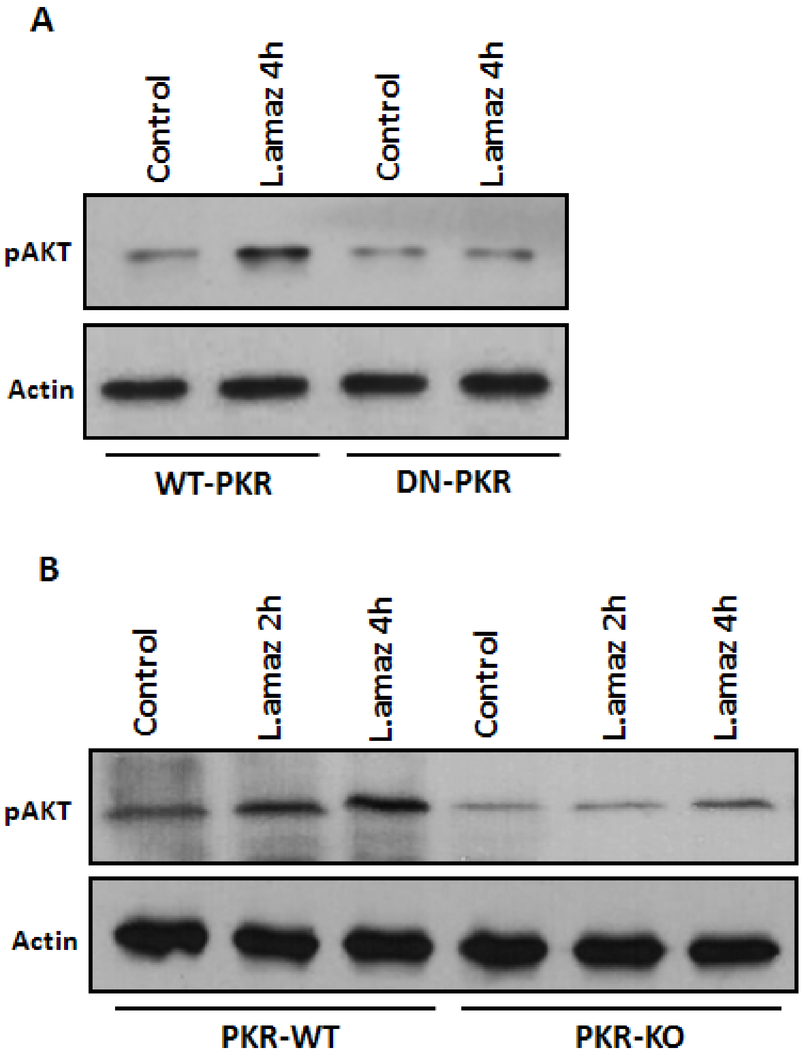

FIGURE S3. *Leishmania amazonensis* induced phosphorylation of Akt1 in a PKR dependent manner.

RAW-WT-PKR or the dominant-negative PKR K296R cells (RAW-DN-PKR) **(A)** or PKR-WT and PKR-KO peritoneal macrophages **(B)** were infected with stationary promastigotes forms of *L. amazonensis* at determinate time and total protein extract were analyzed by western-blot assay with phospho-Akt antibody. Results are representative of 3 independent experiments.

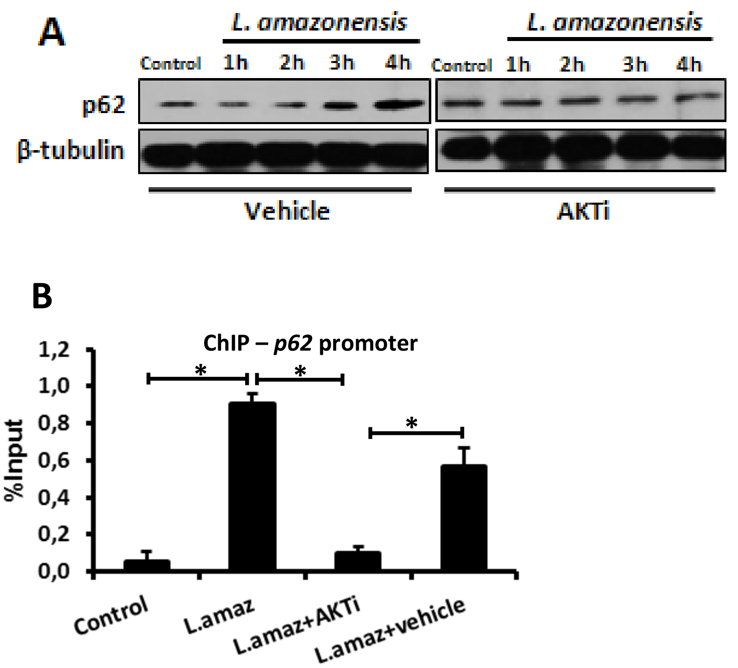

FIGURE S5. p62 regulation by Akt1 signaling.

THP-1 cells were infected with stationary promastigotes forms of *L. amazonensis* and/or treated with Akt-inhibitor-VIII. (A) The total protein extract were analyzed by western-blot assay with p62 antibody. (B) ChIP assay was done using Nrf2 ChIP-antibody and primers for *p62* promoter. Results are representative of 3 independent experiments. *P < 0.05.

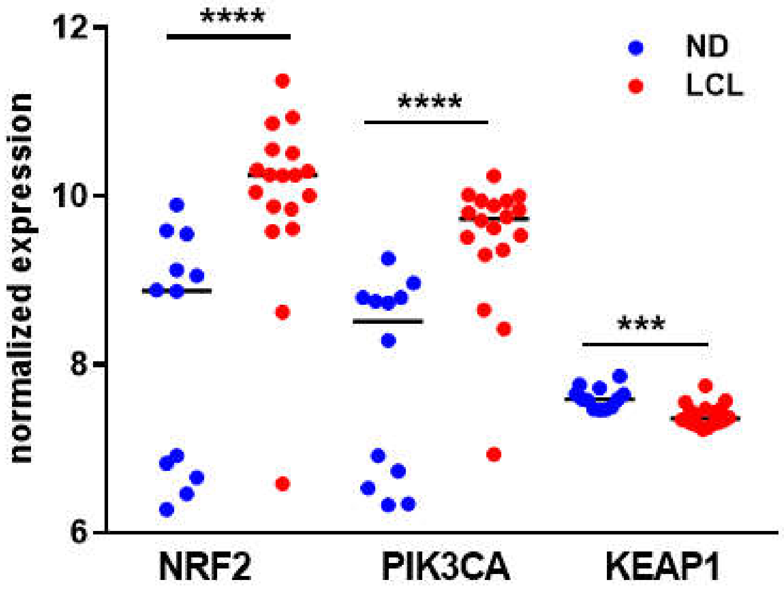

Figure S7| Scatter plot of selected genes significantly up- or down-regulated in PBMC transcriptomes from LCL patients.

Normalized mRNA expression values (Affymetrix Hu Gene 1.0 microarray) are shown for normal donors (n=12) and LCL patients (n=18). Uncorrected p-values(Mann-Whitney) are shown. ***p<0.001, ****p<0.0001.

Gene assignment	Gene symbol	Gene name	Log-ratio	p-value	Corrected p-value
L01664	CLC	Charcot-Leyden crystal protein	2.96	2.17E-06	0.0047
BC009716	CCL2	chemokine (C-C motif) ligand 2	2.69	2.55E-05	0.0167
BC012609	SERPINB2	serpin peptidase inhibitor, clade B (ovalbumin), member 2	2.64	8.15E-05	0.0243
AF117710	HBB	hemoglobin, beta	2.63	1.83E-04	0.0318
AF016495	AQP9	aquaporin 9	2.32	3.09E-05	0.0173
AK292851	NAMPT	nicotinamide phosphoribosyltransferase	2.28	5.00E-04	0.0434
AF368169	B3GNT5	UDP-GlcNAc:betaGal beta-1,3-N-acetylglucosaminyltransferase 5	2.03	9.99E-06	0.0122
BC127088	ZNF267	zinc finger protein 267	2.03	2.40E-05	0.0161
AF430642	GBP5	guanylate binding protein 5	2.01	2.44E-04	0.0346
L10918	CCR1	chemokine (C-C motif) receptor 1	1.92	4.44E-04	0.0408
AF006513	CHD1	chromodomain helicase DNA binding protein 1	1.89	2.78E-04	0.0361
AB011420	STK17A	serinethreonine kinase 17a	1.83	1.66E-04	0.0301
BC032839	IFIT2	interferon-induced protein with tetratricopeptide repeats 2	1.83	2.20E-05	0.0160
**AK297838**	**NFE2L2**	**nuclear factor (erythroid-derived 2)-like 2**	**1.81**	**2.07E-04**	**0.0333**
BC144509	RICTOR	RPTOR independent companion of MTOR, complex 2	1.80	4.36E-04	0.0406
AF097635	HBA1	hemoglobin, alpha 1	1.78	5.35E-04	0.0441
AF097635	HBA1	hemoglobin, alpha 1	1.78	5.35E-04	0.0441
BC028352	TBC1D15	TBC1 domain family, member 15	1.77	1.18E-04	0.0277
AF228422	C15orf48	chromosome 15 open reading frame 48	1.75	2.01E-05	0.0153
AK055599	CTSL1	cathepsin L1	1.71	3.05E-04	0.0370
AK292798	ACSL1	acyl-CoA synthetase long-chain family member 1	1.67	3.50E-04	0.0379
AF287012	GABARAPL1	GABA(A) receptor-associated protein like 1	1.66	2.42E-04	0.0346
BC047891	HCAR3	hydroxycarboxylic acid receptor 3	1.65	2.40E-05	0.0161
ENST00000464839	GBP2	guanylate binding protein 2, interferon-inducible	1.64	1.68E-04	0.0302
BC030205	TNFAIP6	tumor necrosis factor, alpha-induced protein 6	1.64	1.27E-04	0.0281
BC028210	ICOS	inducible T-cell co-stimulator	1.63	7.74E-06	0.0102
U22377	RLF	rearranged L-myc fusion	1.61	2.98E-04	0.0369
**AK292940**	**PIK3CA**	**phosphoinositide-3-kinase, catalytic, alpha polypeptide**	**1.60**	**6.11E-05**	**0.0222**
D42063	RANBP2	RAN binding protein 2	1.57	7.01E-05	0.0230
AB208912	GBP1	guanylate binding protein 1, interferon-inducible	1.57	3.51E-04	0.0379
AF443203	PLIN2	perilipin 2	1.56	2.77E-04	0.0361
AF301016	CXCL16	chemokine (C-X-C motif) ligand 16	1.53	5.45E-04	0.0442
BC098396	MXD1	MAX dimerization protein 1	1.53	2.94E-04	0.0368
AF218085	SAMSN1	SAM domain, SH3 domain and nuclear localization signals 1	1.53	2.68E-04	0.0358
AF070674	BIRC3	baculoviral IAP repeat containing 3	1.50	4.06E-04	0.0400
AB059622	RB1CC1	RB1-inducible coiled-coil 1	1.47	1.32E-04	0.0283
BC018538	ALOX5AP	arachidonate 5-lipoxygenase-activating protein	1.47	3.35E-04	0.0377
ENST00000267953	BCL2A1	BCL2-related protein A1	1.46	9.89E-05	0.0257
AK303625	RASGEF1B	RasGEF domain family, member 1B	1.46	5.15E-04	0.0439
BC035492	MAPK6	mitogen-activated protein kinase 6	1.42	3.50E-04	0.0379
BC130418	ARID4B	AT rich interactive domain 4B (RBP1-like)	1.41	4.04E-04	0.0400
BC017069	HBP1	HMG-box transcription factor 1	1.41	5.76E-04	0.0451
ENST00000439040	ATP13A3	ATPase type 13A3	1.39	6.71E-04	0.0483
AB004550	B4GALT5	UDP-Gal:betaGlcNAc beta 1,4- galactosyltransferase, polypeptide 5	1.39	3.13E-05	0.0173
BX537972	LYSMD3	LysM, putative peptidoglycan-binding, domain containing 3	1.36	4.66E-04	0.0422
AB051232	PTBP2	polypyrimidine tract binding protein 2	1.36	3.18E-05	0.0173

S1 TABLE. Top 50 significantly up-regulated genes in LCL disease signature.

Ingenuity Canonical Pathways	-log (p-value)	Ratio	Molecules
**NRF2-mediated Oxidative Stress Response**	**1.63E00**	**3.89E-02**	**SQSTM1, PIK3CA, DNAJC9, TXNRD1, SCARB1, EIF2AK3, NFE2L2**
Thioredoxin Pathway	1.04E00	1.67E-01	TXNRD1
Antioxidant Action of Vitamin C	3.47E-01	2.06E-02	CSF2RB, TXNRD1

S2 TABLE: Antioxidant pathways upregulated in the LCL disease signature.

Characteristic	LCL patients	DCL patients
Gender M : F	3: 2	3:1
Age, mean ± SD (range), years	66.6 ± 19.5	17.57 ± 13.13
DTH positive	4 : 1	0
No. of active lesions, mean (range)	1.2 ± 0.4	138.4 ± 169.2
Time of disease (months)	2.75 ± 1.7	190.3 ± 80.92

S3 TABLE. Epidemiological and clinical parameters for LCL and DCL patients.

NOTE: LCL, Localized cutaneous leishmaniasis; DCL, Diffuse cutaneous leishmaniasis; M, male; F, female; DTH, delayed hypersensitivity skin-test response.
